# Pyrimidine Metabolic Dysregulation Following *pyrR* Knockout Attenuates the Anti-Biofilm, Anti-Inflammatory, and Antioxidant Activities of *Lacticaseibacillus casei* Cell-Free Supernatant

**DOI:** 10.3390/biology15211914

**Published:** 2026-10-22

**Authors:** Yaokun Jiang, Fengze Han, Xinmiao He, Shihan Zhao, Meiqin Sun, Libing Zhang, Kunzhi Yu, Yuxuan Zhao, Ziluo Zou, Wentao Wang, Heshu Chen, Xinpeng Jiang, Di Liu

**Affiliations:** 1 Key Laboratory of Combining Farming and Animal Husbandry, Ministry of Agriculture, Animal Husbandry Research Institute, Heilongjiang Academy of Agricultural Sciences, No. 368 Xuefu Road, Harbin 150086, China; haashxm@163.com (X.H.); wangwentao@haas.cn (W.W.); chenheshu@haas.cn (H.C.); 2 College of Animal Science and Technology, Northeast Agricultural University, Harbin 150030, China; jyk19947898605@outlook.com (Y.J.); 15945905766@163.com (F.H.); 15532667681@163.com (S.Z.); s15124450525@163.com (M.S.); 16696089886@163.com (L.Z.); 15965068275@163.com (K.Y.); z2167370935@163.com (Y.Z.); 18571850012@163.com (Z.Z.)

**Keywords:** *Lacticaseibacillus casei*, pyrimidine, pyrimidine metabolic dysregulation, anti-biofilm

## Abstract

Pathogenic infections caused by intestinal microecological imbalance pose a critical threat to livestock breeding and human health. Multiple probiotic functions of *Lacticaseibacillus casei* (*L. casei*), an important probiotic, are modulated by *pyrR*-mediated pyrimidine metabolism. This study used *L. casei* isolated from the intestines of Min pigs (a local pig breed from northeastern China) and its *pyrR* gene knockout mutant (named *L. casei* Δ*pyrR*, Δ*pyrR*) to investigate their antibacterial, anti-inflammatory, and antioxidant capacities. The results showed that the mutant exhibited significantly attenuated effects compared with the wild-type in disrupting pre-formed biofilms, damaging bacterial morphology, and downregulating the expression of related genes (biofilm and flagellar genes) in *Staphylococcus aureus* (*S. aureus*) and *Salmonella typhimurium* (*S. typhimurium*). The mutant-derived supernatant also exhibited impaired anti-inflammatory effects in *S. typhimurium*-challenged IPEC-J2 cells: it was less effective at restraining pathogen-induced IL-1β upregulation and boosting the production of host-derived TGF-β and IL-10, accompanied by altered regulation of the host STING signaling pathway. However, existing data cannot fully distinguish whether STING alteration corresponds to suppressed inflammation or impaired immunity. Additionally, pyrimidine metabolism in *L. casei* suppresses pathogen-induced reactive oxygen species (ROS) accumulation. These findings are consistent with *pyrR*-mediated pyrimidine synthesis contributing to the probiotic functions of *L. casei*, providing an experimental basis for optimizing probiotic preparations and substituting antibiotics.

## Simple Summary

Intestinal infections caused by harmful bacteria such as *Staphylococcus aureus* and *Salmonella* remain a serious threat to animal farming and human health, and overusing antibiotics to fight them has driven widespread drug resistance. Probiotics are friendly bacteria, such as *Lacticaseibacillus casei*, which naturally live in the gut and offer a promising alternative, but how their internal chemistry shapes their benefits is poorly understood. Here, researchers removed a regulatory gene called *pyrR* from a strain of *Lacticaseibacillus casei* originally isolated from pig intestine and compared it with the normal bacterium. The modified bacterium became much weaker: its culture fluid was less able to stop harmful bacteria from building protective slimy layers called biofilms, to damage bacterial cells, and to silence the genes that make them virulent. In pig intestinal cells challenged with *Salmonella*, it also did a poorer job of calming inflammation and lowering harmful oxygen molecules that stress cells. Adding back a simple building block called uracil partly restored these benefits. These findings show that normal pyrimidine chemistry inside this probiotic is essential for it to work well, giving scientists a clearer blueprint for designing better probiotic supplements and reducing reliance on antibiotics in animal farming.

## 1. Introduction

*Lacticaseibacillus casei* is a common Gram-positive bacterium with probiotic functions, which can be isolated from different parts of the human body and the intestines of animals [1]. It is an important component of the normal gut microbiota of humans and animals and plays a significant role in maintaining animal health. Recombinant *L. casei* expressing the K88 fimbrial protein gene of enterotoxigenic *Escherichia coli* (ETEC) modulates the intestinal probiotic microbiota and effectively reduces the diarrhea rate in newborn piglets [2]. Feeding *L. casei* Zhang can prevent jejunal injury caused by *E. coli* K88 in early-weaned piglets [3]. *L. casei* mainly supports the health of the intestinal microbiota by competitively inhibiting the proliferation of harmful intestinal bacteria, modulating its structural balance, and promoting the production of beneficial metabolic products (such as short-chain fatty acids). The bacteriocin-like inhibitory substance (BLS) produced by *Lactobacillus* spp. has broad-spectrum antibacterial properties and can inhibit both Gram-positive bacteria (such as *S. aureus*) and Gram-negative bacteria (such as *S. typhimurium*) [4]. The cell-free supernatant (CFS) of *Lacticaseibacillus rhamnosus* SCB0119 inhibits the growth of *Escherichia coli* and *S. aureus* and induces bacterial death through both physiological damage and molecular regulatory mechanisms [5].

The antibacterial ability of lactic acid bacteria (LAB) is significantly influenced by their growth environment and metabolic level. As a core branch of nucleotide metabolism, pyrimidine metabolism plays multifaceted roles in supporting the growth, stress resistance and environmental adaptation of LAB [6,7]. Under high-density culture conditions, *Lactobacillus paracasei* B1 not only provides bases for the DNA repair process itself through pyrimidine and purine metabolism, but also produces metabolic intermediates that induce and promote DNA repair, helping bacteria cope with environmental stress and increasing the survival rate of the strain [8]. At the molecular level, pyrimidine biosynthesis in multiple Gram-positive bacteria is regulated by the PyrR protein, a conserved pyrimidine-responsive regulatory protein [9]. PyrR functions as an RNA-binding attenuation protein that specifically binds to the 5′ leader sequence of the *pyr* operon mRNA [10]. When intracellular pyrimidine nucleotides are abundant, PyrR binding induces transcriptional termination upstream of the pyrimidine biosynthetic structural genes and represses pyrimidine synthesis in vivo [11]. When pyrimidine levels are insufficient, PyrR dissociates from the mRNA, allowing full transcription of the *pyr* operon to replenish the pyrimidine pool [11]. Genetic disruption of *pyrR* therefore disturbs the homeostatic regulation of pyrimidine metabolism, leading to profound alterations in the intracellular and extracellular profiles of pyrimidine metabolites, which in turn affect multiple physiological phenotypes of the strain [10].

In our previous study, we deleted the *pyrR* gene in *L. casei* through CRISPR-Cas9 genome editing and constructed a pyrimidine metabolism-disrupted mutant strain designated *L. casei* Δ*pyrR* (Δ*pyrR*) [12]. Non-targeted LC-MS metabolomics of this mutant confirmed marked depletion of pyrimidine-related and secondary antibacterial metabolites in its culture supernatant, and the direct bacteriostatic activity of the Δ*pyrR* mutant against *S. aureus* and *S. typhimurium* was significantly attenuated compared with that of the wild-type *L. casei* [12]. However, these initial findings were limited to the direct growth-inhibitory effect of the strain. It remains unknown whether *pyrR*-mediated pyrimidine metabolism also modulates other probiotic functions of *L. casei*, particularly its anti-biofilm activity against pathogens, its anti-inflammatory and antioxidant effects on host intestinal epithelial cells, and the underlying phenotypic and molecular mechanisms. Specifically, it is unclear whether pyrimidine metabolic dysregulation affects the ability of *L. casei* to disrupt pathogen morphological integrity, downregulate biofilm- and virulence-associated gene expression in pathogens, and alter host innate immune signaling and oxidative stress responses in intestinal epithelial cells (IPEC-J2 cells).

To address these knowledge gaps, we compared the wild-type *L. casei* and its Δ*pyrR* mutant for their effects on pre-formed biofilms, cellular morphology, and the expression of biofilm- and flagellum-related genes in *S. aureus* and *S. typhimurium*, and further assessed their anti-inflammatory and antioxidant activities in *S. typhimurium*-challenged IPEC-J2 cells. This study aims to explore the involvement of pyrimidine metabolism in the probiotic properties of *L. casei*, and to provide experimental data for subsequent mechanistic investigations.

## 2. Materials and Methods

### 2.1. Bacterial Strains

The wild-type *L. casei* strain and its *pyrR* gene knockout mutant (*L. casei* Δ*pyrR*) used in this study are identical to the strains described in our previous study [12], in which the *L. casei* strain was isolated from the intestine of Min pigs. Both strains were inoculated separately into MRS broth (Qingdao Hope Bio-Technology Co., Ltd., Qingdao, China) at an initial optical density (OD_600_) of 0.05 and incubated statically at 37 °C for 16–18 h. The culture was terminated when the OD_600_ reached 0.8 to ensure that the bacteria were in the late logarithmic phase to early stationary phase. The two strains entered the stationary phase between 16 and 18 h, and at 18 h, their log_10_ CFU/mL values were comparable [12]. For the exogenous uracil (Sigma-Aldrich Merck KGaA, Darmstadt, Germany) complementation assay (Section 2.3), filter-sterilized uracil stock solution was added to MRS broth to a final concentration of 20 μg/mL before inoculation. *L. casei* Δ*pyrR* was then inoculated into the uracil-supplemented MRS broth following the same protocol described above. *Staphylococcus aureus* and *S. typhimurium* were inoculated into LB broth separately, and cultured at 37 °C with shaking at 200 rpm for 12 h. LB broth (10 g/L tryptone, 5 g/L yeast extract, 10 g/L NaCl) was prepared in-house and autoclaved at 121 °C for 15 min. The *S. aureus* (CMCC 26003) and *Salmonella typhimurium* (ATCC 14028) strains used in this study were preserved in our laboratory. The original *S. aureus* strain was purchased from the National Center for Medical Culture Collections (CMCC), while the original *Salmonella typhimurium* strain was purchased from the American Type Culture Collection (ATCC). The IPEC-J2 cells (RRID: CVCL_2246; DSMZ ACC 701) used in this study are maintained in our laboratory, and the original cell stock was purchased from Tong Pai Technology Co., Ltd. (Shanghai, China).

### 2.2. Preparation of the Supernatant of Lacticaseibacillus casei

The *L. casei*, *L. casei* Δ*pyrR*, and uracil-supplemented *L. casei* Δ*pyrR* strains were cultured following the procedures described in Section 2.1 [12]. The suspensions of all three strains were subjected to centrifugation at 12,000× *g* for 10 min, and the supernatants were filtered through a 0.22-µm membrane filter to remove bacterial cells. The sterility of the supernatants was evaluated by streaking onto nutrient agar plates [13]. After centrifugation and filtration, the pH values of the cell-free supernatants from *L. casei* and *L. casei* Δ*pyrR* were measured (4.3 ± 0.1 for *L. casei* and 5.1 ± 0.1 for *L. casei* Δ*pyrR*; n = 3). For IPEC-J2 cell assays, the supernatants were adjusted to pH 7.2 ± 0.05 with sterile NaOH, and then re-filtered through a 0.22-µm membrane filter. For the pathogen-related assays (disruption of pre-formed biofilms, TEM, and RT-qPCR), the supernatants were adjusted to pH 5.5 ± 0.1 [14] with sterile NaOH and re-filtered through a 0.22-µm membrane filter prior to use. The resulting supernatants were then stored at −20 °C for subsequent experimental use.

### 2.3. Evaluation of the Disruptive Effect of L. casei Cell-Free Supernatant on Pre-Formed Biofilms

*Staphylococcus aureus* and *S. typhimurium* were cultured following the procedures described in Section 2.1, then diluted to 5 × 10^6^ CFU/mL. Subsequently, the pathogenic microorganism suspensions were separately inoculated into 96-well flat-bottom plates [15]. Plates were statically cultured at 37 °C for 6 h to facilitate the initial adhesion and formation of stable pathogenic biofilms [16]. Non-adherent bacterial cells were removed by gentle aspiration of the culture medium, followed by two gentle rinses with sterile PBS [16]. To each well of the plates, except those in the control group, 100 µL of sterile LB broth was added. Then, 100 µL of supernatant from *L. casei*, *L. casei* Δ*pyrR*, and uracil-supplemented *L. casei* Δ*pyrR* strains, diluted in LB broth, was added separately. For the uracil complementation group, the supernatant was prepared from *L. casei* Δ*pyrR* cultured in MRS broth supplemented with 20 μg/mL uracil as described in Section 2.1. All cell-free supernatants were diluted using the same volumetric protocol (1:64, 1:128, 1:256, 1:512, and 1:1024, *v*/*v*). A preliminary experiment further confirmed that sterile MRS broth, prepared at the same dilution ratios as used in the formal experiment but without bacterial inoculation, did not significantly affect biofilm formation by *S. aureus* or *S. typhimurium*, and that uracil alone at the tested concentration (20 μg/mL) had no significant effect on biofilm formation by either pathogen under the experimental conditions. In the control group, only 200 µL of LB broth was added. All of the 96-well plates were statically incubated at 37 °C for 24 h. After incubation, the medium was carefully aspirated, and the wells were washed thoroughly with sterile PBS. The 96-well plates were placed in a drying oven at 60 °C for 30 min to fix the cells [17]. Then, 0.1% CV (*w*/*v*) crystal violet staining solution was added to each well, and the plates were incubated for 15 min [18]. 33% (*v*/*v*) glacial acetic acid was added to each well to decolorize the wells for 10 min, and then the optical density (OD) was determined at 590 nm to quantify biofilm biomass using an ELx800 microplate reader (BioTek, Winooski, VT, USA). The underlying OD_590_ values are provided in Table S1.

### 2.4. Observation of S. aureus and S. typhimurium Under the Influence of Lacticaseibacillus casei by Transmission Electron Microscopy

*Staphylococcus aureus* and *S. typhimurium* were cultured following the procedures described in Section 2.1, then separately co-cultured with the supernatants of *L. casei* and *L. casei* Δ*pyrR* for 12 h. The minimum inhibitory concentration (MIC) of the *L. casei* supernatant against each pathogen was predetermined through the standard broth microdilution method (described in the Supplementary Materials), defined as the lowest supernatant dilution ratio that completely inhibits visible bacterial growth after 24 h of incubation. The concentrations of the *L. casei* supernatant used were MIC, 1/2 MIC, and 1/4 MIC against the two pathogenic microorganisms [19,20]. For the *L. casei* Δ*pyrR* group, the supernatant was added at exactly the same volume dilution ratios as those of the *L. casei* supernatant. In the control group, *S. aureus* and *S. typhimurium* were cultured using only LB broth. Then, the samples were centrifuged at 4 °C and 3000× *g* for 15 min and the supernatants were subsequently removed. The samples were washed once with sterile PBS, then fixed with 2.5% glutaraldehyde followed by post-fixation with 1% osmium tetroxide for 1 h at room temperature. Subsequently, dehydration was performed using a graded ethanol series of increasing concentrations [21]. Finally, the samples were examined under a transmission electron microscope (TEM, Hitachi HT7820 Hitachi High-Tech Corporation, Tokyo, Japan), and images were recorded. For each experimental condition, at least 5 fields of view were observed, and representative images were selected to reflect the predominant morphological phenotype of the bacterial population. The original image is shown in Figure S1.

### 2.5. Quantification of Gene Expression Using RT-qPCR

*Staphylococcus aureus* and *S. typhimurium* were cultured at 37 °C and 180 rpm until the OD_600_ value reached 0.6. Then, the supernatants of *L. casei* and *L. casei* Δ*pyrR* were added respectively, while the control group was supplemented with LB broth. The cultures were further incubated at 37 °C and 200 rpm for 12 h. The bacterial suspensions of *S. aureus* and *S. typhimurium* from each group were collected by centrifugation, and three independent RNA samples were extracted. RNA was extracted and purified using the TransZol Up Kit (TransGen Biotech, Beijing, China). The EasyScript First-Strand cDNA Synthesis Super Premix Kit (TransGen Biotech, Beijing, China) was used for cDNA synthesis [22]. The expression of biofilm-related genes (*cidA*, *icaA*, *dltB*, *agrA*, *srtA* and *sarA*) in *S. aureus,* as well as that of biofilm- and flagellum-related genes (*glgC*, *fimF*, *fliF* and *lpfA*) in *S. typhimurium* was analyzed by RT-qPCR. The specific primers for *S. aureus* biofilm-related genes and for the *16S rRNA* gene (used as a housekeeping gene), as well as the specific primers for *S. typhimurium* biofilm-related genes and for the *gyrA* gene (used as a housekeeping gene), are listed in Table 1 [23,24]. RT-qPCR was performed in triplicate on the StepOne™ Real-Time PCR system (Applied Biosystems, Foster City, CA, USA) using the SYBR Green Master mix (TransGen Biotech, Beijing, China). The expression levels of the relevant genes were analyzed using the 2^−ΔΔCT^ method [25]. Prior to formal experiments, all primers were validated via standard curves for amplification efficiency (90–110%, R^2^ > 0.98) and melt curve analysis to confirm single specific amplicons without primer dimers. The stability of the reference genes (*16S rRNA gene* for *S. aureus* and *gyrA* for *S. typhimurium*) was validated under the experimental conditions by comparing their cycle threshold (Ct) values across all treatment groups; no significant differences in Ct values were observed among groups (*p* > 0.05, one-way ANOVA), confirming their suitability as endogenous controls in this study.

### 2.6. Western Blot

The IPEC-J2 cell line was used as the in vitro model in this study [26]. Cells were routinely cultured in DMEM/F-12 medium (Dulbecco’s Modified Eagle Medium/Nutrient Mixture F-12, Thermo Fisher Scientific, Waltham, MA, USA) supplemented with 10% (*v*/*v*) fetal bovine serum (Gibco Thermo Fisher Scientific, Waltham, MA, USA) and 1% (*v*/*v*) penicillin–streptomycin solution (100 U/mL penicillin, 100 μg/mL streptomycin, Gibco Thermo Fisher Scientific, Waltham, MA, USA). All cell cultures were maintained at 37 °C in a humidified atmosphere with 5% CO_2_, and the medium was replaced every 48 h. When cells reached 80–90% confluence, they were passaged using 0.25% trypsin-EDTA (Gibco Thermo Fisher Scientific, Waltham, MA, USA) digestion. For formal experiments, cells were seeded at the target density and cultured overnight to allow complete adherence before treatment. The same cell culture conditions were applied for the ROS detection assay described in Section 2.7.

For the infection experiments, confluent IPEC-J2 monolayers were washed twice with sterile PBS and pre-incubated in antibiotic-free DMEM/F-12 for 30 min to eliminate residual penicillin–streptomycin prior to bacterial challenge [27]. *Salmonella typhimurium* was harvested by centrifugation (4000× *g*, 10 min), washed twice with sterile PBS, and resuspended in antibiotic-free DMEM/F-12. The bacterial suspension was added to the cells at a multiplicity of infection (MOI) of 10:1 (1 × 10^7^ CFU/mL) [27,28]. The cell-free supernatant was added at a final concentration of 10% (*v*/*v*) of the culture volume immediately before bacterial addition (co-treatment), and the cells were co-cultured with *S. typhimurium* for 8 h at 37 °C in a 5% CO_2_ atmosphere. Untreated control cells received an equal volume of antibiotic-free DMEM/F-12. After treatment, the medium was removed, and the cells were washed three times with sterile PBS; fresh DMEM/F-12 containing 1% (*v*/*v*) penicillin–streptomycin was then added to terminate further bacterial growth [27]. The same infection and treatment procedures were applied to all assays (Western blot; ROS).

The IPEC-J2 cells were divided into the following treatment groups. *L. casei* group: IPEC-J2 cells were co-cultured with *L. casei* cell-free supernatant for 8 h. *L. casei* Δ*pyrR* group: IPEC-J2 cells were co-cultured with *L. casei* Δ*pyrR* cell-free supernatant for 8 h. *Salmonella typhimurium* group: *S. typhimurium* suspension was added to IPEC-J2 cells and co-cultured for 8 h. *L. casei* and *S. typhimurium* group: supernatant of *L. casei* was added to IPEC-J2 cells, followed by the addition of *S. typhimurium* suspension, and co-cultured for 8 h. *L. casei* Δ*pyrR* and *S. typhimurium* group: supernatant of *L. casei* Δ*pyrR* was added to IPEC-J2 cells, followed by the addition of *S. typhimurium* suspension, and co-cultured for 8 h.

Cells were lysed in RIPA buffer containing protease inhibitors (Beyotime Biotechnology, Shanghai, China) on ice for 30 min, and lysates were clarified by centrifugation at 12,000× *g* for 10 min at 4 °C. Protein concentration was determined using the Bradford assay (Nanjing Jiancheng Bioengineering Institute, Nanjing, China) to ensure equal loading of proteins in SDS-PAGE. Protein samples (20 μg) were separated on 12% SDS-PAGE gels (80 V for 30 min in the stacking gel, followed by 120 V for 1 h in the resolving gel) and transferred onto PVDF membranes (Bio-Rad Laboratories, Hercules, CA, USA), followed by immunoblotting with the following primary antibodies: STING (1:5000), IL-1β (1:3000), TGF-β (1:3000), IL-10 (1:3000) and β-actin (1:5000) (ABclonal, Inc., Woburn, MA, USA). According to the manufacturers’ specifications, these antibodies recognize the mature forms of IL-1β (~17 kDa), TGF-β (~25 kDa, mature dimer), and IL-10 (~37 kDa, mature dimer). The secondary antibodies included horseradish peroxidase-labeled goat anti-rabbit and mouse antibodies (1:500) (ABclonal, Inc., Woburn, MA, USA). Finally, the PVDF membrane was exposed using ECL luminescent solution (Beyotime Biotechnology, Shanghai, China) through the Western blot Molecular Imaging System 300 (Azure Biosystems, Dublin, CA, USA) [29]. The gray values of the bands and the internal reference were measured using ImageJ/Fiji (version 2.9.0/1.54f, National Institutes of Health, Bethesda, MD, USA). The relative expression level of each target protein was calculated by normalizing its band intensity to that of the internal reference β-actin. All quantitative results were derived from three independent biological replicates. Original Western blot images are provided in Figure S2.

In this study, the same IPEC-J2 cell line was used for both Western blot and ROS detection assays (Section 2.7). Preliminary experiments demonstrated that MRS broth did not induce cellular oxidative stress or inflammatory activation. The background effect of MRS broth was uniformly controlled across all supernatant treatment groups. Preliminary experiments further confirmed that pure MRS broth exerted no significant effect on the basal expression levels of IL-1β, STING, TGF-β, and IL-10. In the Western blot experiment, the *S. typhimurium*-only treatment group served as the positive control to reflect pathogen-induced inflammatory responses.

### 2.7. Assays for Reactive Oxygen Species

IPEC-J2 cells were seeded in 6-well plates, and DCFH-DA (Beyotime Biotechnology, Shanghai, China) was added to the cultured IPEC-J2 cells at a final concentration of 10 µM, and the cells were incubated in the dark at 37 °C for 20 min without harvesting the cells [30]. The level of ROS was detected by fluorescence microscopy (Nikon Corporation, Tokyo, Japan), and the relative fluorescence intensity was quantified using ImageJ/Fiji software.

### 2.8. Statistical Analysis

The absorbance values were measured using an ELx800 microplate reader. ImageJ/Fiji software was used for the quantitative analysis of the gray values of Western blot bands and the relative fluorescence intensity. The expression of related genes was analyzed by the 2^−ΔΔCT^ method. All data are presented as the mean ± standard deviation (SD) of three independent biological replicates (n = 3). The biofilm disruption assay (Section 2.3) served as a semi-quantitative preliminary screen and was presented descriptively without formal statistical testing. One-way analysis of variance (ANOVA) was applied to the statistical analysis of the remaining measurement indices. Post-ANOVA pairwise comparisons among groups were performed using Tukey’s multiple comparison test [31]. All statistical analyses were performed using GraphPad Prism software (Version 10.5.0, GraphPad Software, San Diego, CA, USA). Each experiment was performed in triplicate as independent biological replicates. A *p*-value < 0.05 was considered statistically significant. For all image-based quantitative assays, formal single-blinded assessment was not applied in this in vitro study. To minimize potential subjective bias, all image acquisition and quantification were independently performed by two trained researchers following pre-set uniform instrument parameters and standardized ImageJ/Fiji analysis protocols. All measurement thresholds and analysis regions were fixed across all experimental groups without manual or subjective adjustment.

## 3. Results

### 3.1. Deletion of the pyrR Gene Reduces the Anti-Biofilm Activity of L. casei Against S. aureus and S. typhimurium, While Exogenous Uracil Supplementation Partially Restores This Defective Phenotype

**Figure 1:**
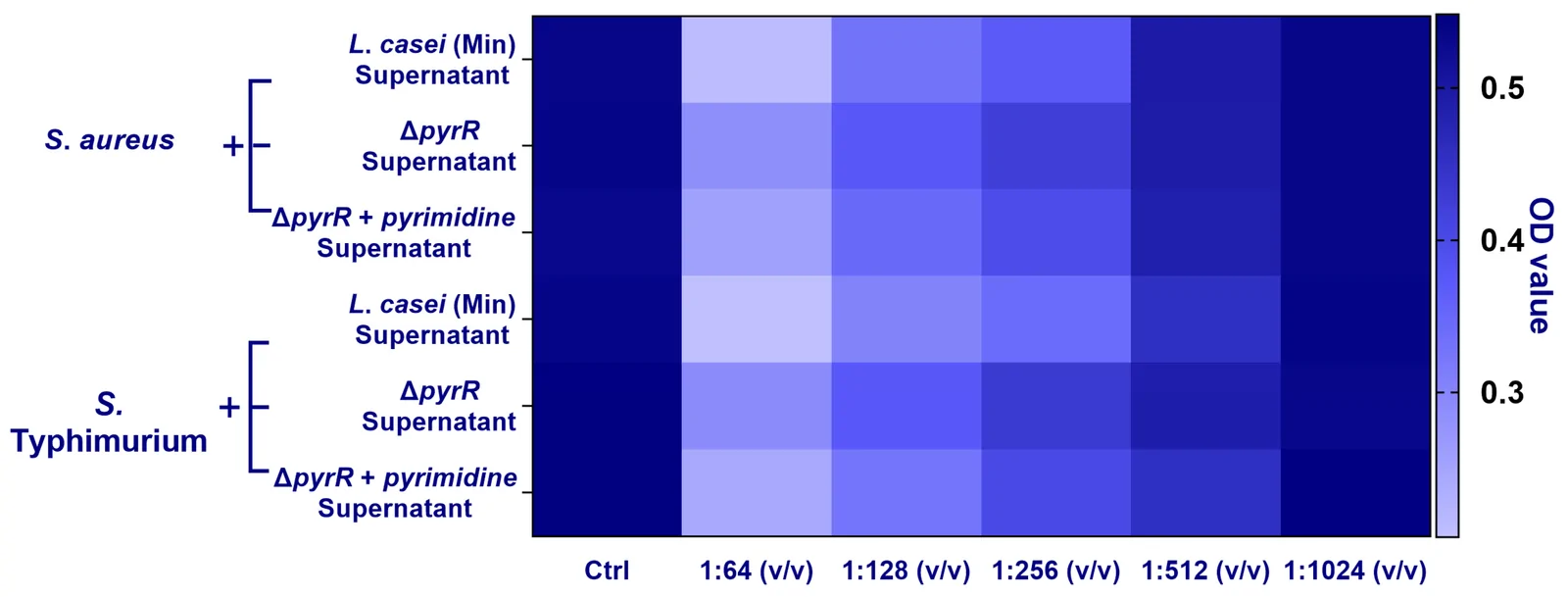
Pathogens were cultured in LB medium with supernatant diluted at 1:64, 1:128, 1:256, 1:512, and 1:1024 (*v*/*v*); the blank control group was treated with pure LB medium. Three treatment groups were included: *L. casei* supernatant, *L. casei* Δ*pyrR* supernatant, and supernatant from *L. casei* Δ*pyrR* cultured with 20 μg/mL exogenous uracil. Biofilm biomass was stained with 0.1% crystal violet and decolorized with 33% glacial acetic acid, and absorbance was determined at OD_590_ using an ELx800 microplate reader. Higher OD_590_ values correspond to denser biofilm formation, as indicated by the color gradient scale bar. All data represent the mean values of three independent biological replicates (n = 3). This heat map illustrates the overall trend of biofilm biomass; as a preliminary semi-quantitative screen, no formal statistical test was applied to this assay.

The supernatants of *L. casei* (Min) and *L. casei* Δ*pyrR*, and uracil-supplemented *L. casei* Δ*pyrR* were diluted at different ratios and co-cultured with *S. aureus* and *S. typhimurium*. The control group was only treated with LB broth. The results of crystal violet staining are shown in Figure 1 with the heat map. Darker color indicates higher absorbance and more biofilm formation. We observed that co-culture with supernatants from either wild-type *L. casei* or *L. casei* Δ*pyrR* reduced the absorbance of *S. aureus* and *S. typhimurium* relative to the control, indicating that both supernatants reduced the biomass of adherent biofilms formed by these two pathogens under the tested conditions. Moreover, the absorbance of *S. aureus* and *S. typhimurium* co-cultured with the supernatant of *L. casei* Δ*pyrR* was consistently higher than that of cultures co-cultured with the supernatant of *L. casei*, indicating that the anti-biofilm effect of the *L. casei* Δ*pyrR* supernatant against these two bacteria was attenuated compared with that of the *L. casei* supernatant.

To further confirm that the attenuated anti-biofilm activity was caused by pyrimidine metabolic disruption resulting from the *pyrR* knockout, we prepared cell-free supernatant from *L. casei* Δ*pyrR* cultured with exogenous uracil at a final concentration of 20 μg/mL and evaluated its disruptive capacity on pre-formed biofilms. The results showed that exogenous uracil supplementation restored the anti-biofilm effect of the *L. casei* Δ*pyrR* supernatant against both *S. aureus* and *S. typhimurium* across all tested dilution ratios. Compared with the non-supplemented *L. casei* Δ*pyrR* group, the biofilm biomass of both pathogens was decreased after treatment with uracil-supplemented Δ*pyrR* supernatant. However, the anti-biofilm efficacy of the 20 μg/mL uracil complementation group did not fully reach the level of the *L. casei* supernatant.

### 3.2. Morphological Alterations in S. aureus and S. typhimurium Induced by Cell-Free Supernatants from L. casei and L. casei ΔpyrR

**Figure 2:**
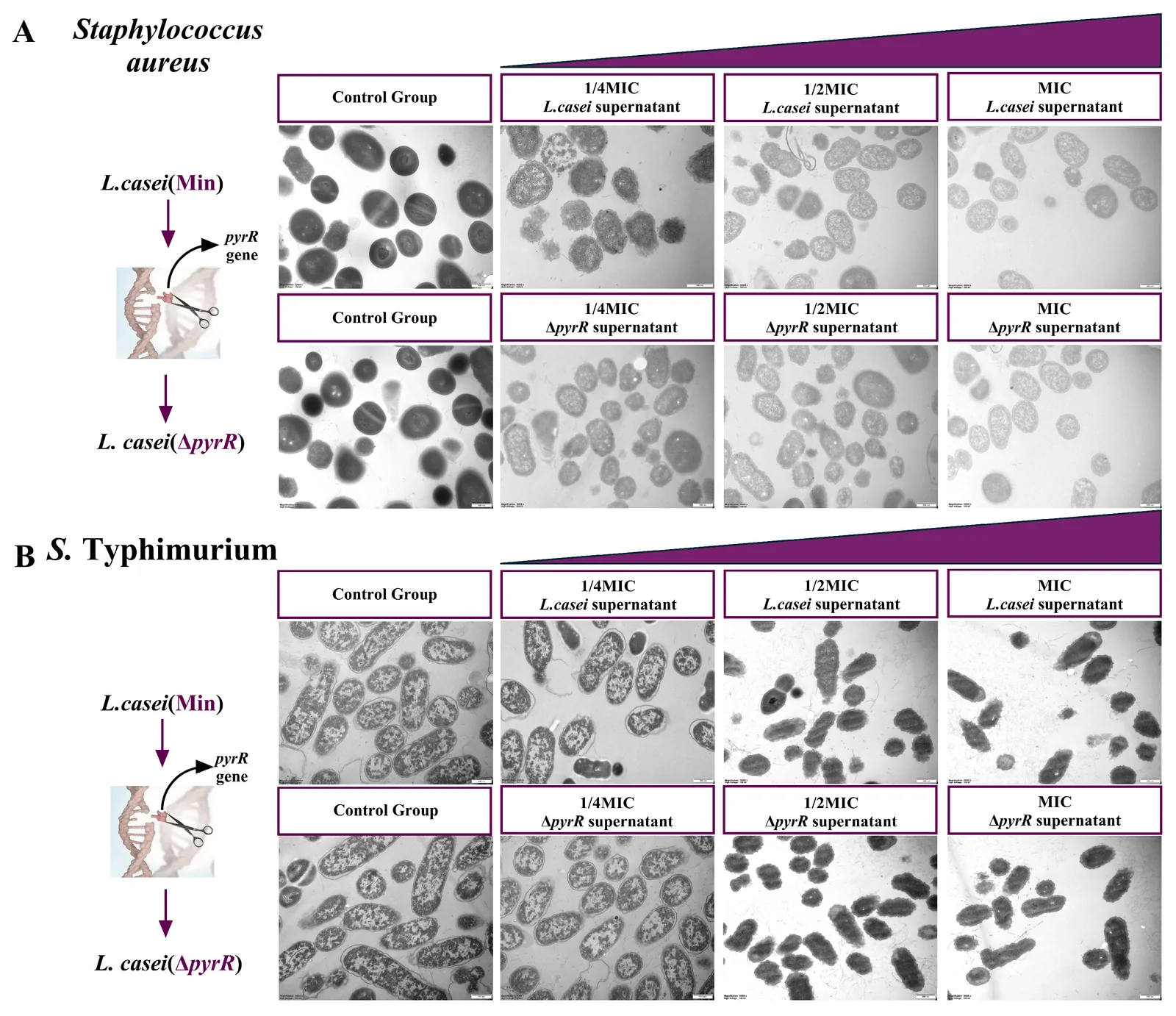
Transmission electron microscopy (TEM) images of *S. aureus* (**A**) and *S. typhimurium* (**B**) co-cultured with *L. casei* and *L. casei* Δ*pyrR* supernatant. The control groups were with LB broth. Changes in the morphology of the pathogenic microorganisms were observed upon treatment with different concentrations of *L. casei* and *L. casei* Δ*pyrR* supernatants. The scale bar for all images is 500 nm.

To explore the effects of *L. casei* (Min) and *L. casei* Δ*pyrR* supernatants on the morphological characteristics of *S. aureus* (Figure 2A) and *S. typhimurium* (Figure 2B), the morphological features of the bacteria were observed by transmission electron microscopy. When *S. aureus* and *S. typhimurium* were treated with the supernatant of *L. casei* at the minimum inhibitory concentration (MIC), membrane disruption was observed, with pore-like damage and leakage of intracellular contents visible in the representative images; *S. typhimurium* cells also appeared rounded, with flagella detached from the cell surface. As the concentration of the *L. casei* supernatant decreased, membrane disruption and intracellular content leakage appeared less pronounced. At the MIC, membrane damage was observed in a smaller proportion of cells treated with the *L. casei* Δ*pyrR* supernatant, and intracellular content leakage appeared reduced in *S. typhimurium*. At lower concentrations, the extent of morphological disruption in the *L. casei* Δ*pyrR* group appeared less pronounced than that in the *L. casei* (Min) group. The morphological features of *S. aureus* were similar to those of *S. typhimurium* upon treatment with the *L. casei* supernatant. Overall, based on the representative images, the *L. casei* supernatant appeared to induce more extensive membrane disruption and content leakage in both pathogenic microorganisms than did the *L. casei* Δ*pyrR* supernatant at the corresponding concentrations.

### 3.3. Altered Expression of Biofilm-Associated Genes in S. aureus and S. typhimurium Induced by Cell-Free Supernatants from L. casei and L. casei ΔpyrR

**Figure 3:**
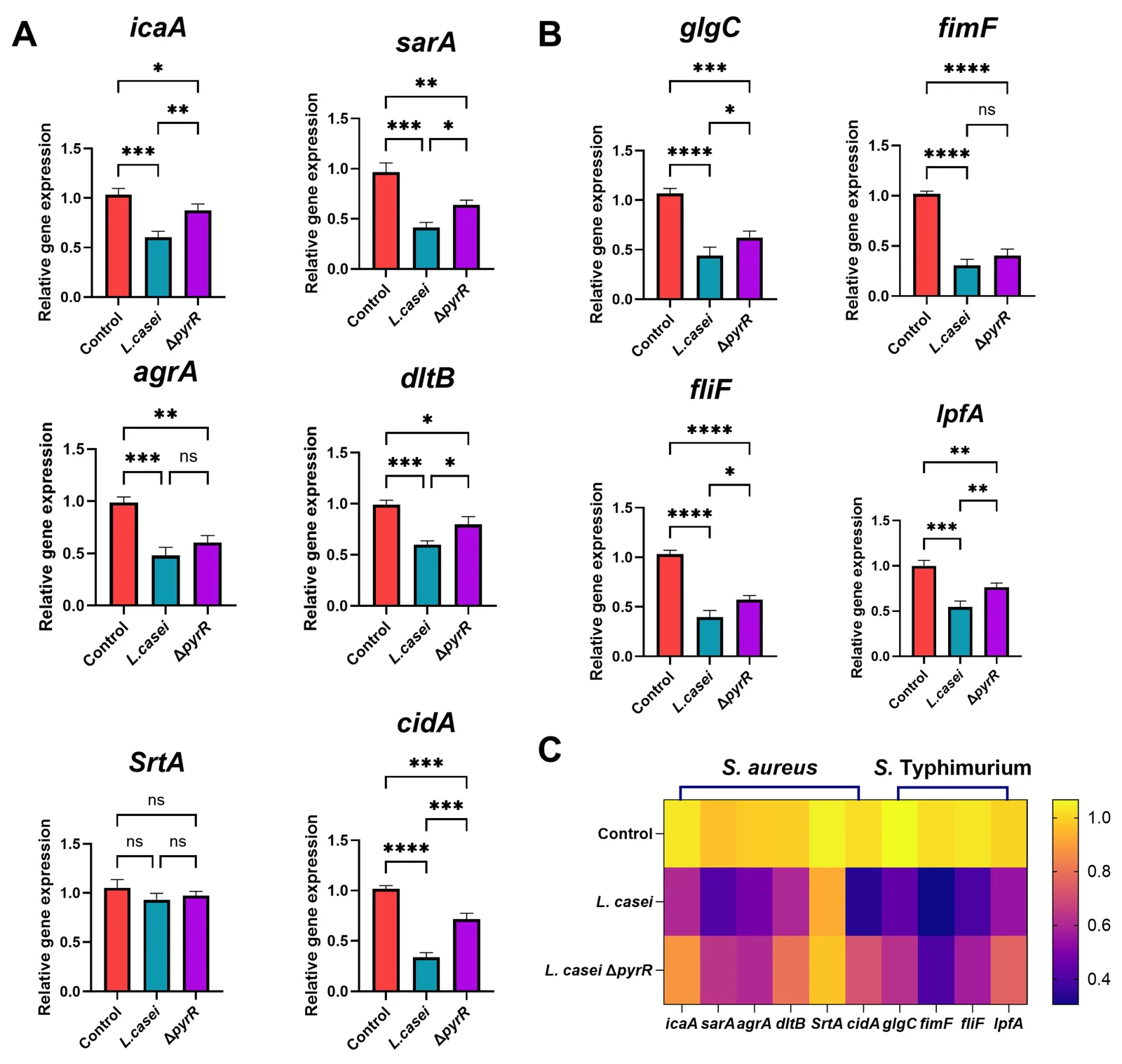
Relative gene expression was calculated using the 2^−ΔΔCT^ method, with *16S rRNA gene* and *gyrA* serving as the endogenous reference genes for *S. aureus* (**A**) and *S. typhimurium* (**B**), respectively. A heatmap summarizing fold changes across all genes (**C**). All data are presented as mean ± standard deviation (SD) from three independent biological replicates (n = 3). Statistical significance was determined by one-way ANOVA followed by Tukey’s multiple comparison test. ns, not significant (*p* > 0.05); *, *p* < 0.05; **, *p* < 0.01; ***, *p* < 0.001; ****, *p* < 0.0001.

Real-time quantitative PCR (RT-qPCR) was performed to quantify the expression of biofilm- and flagellum-related genes in *S. aureus* and *S. typhimurium* following exposure to cell-free supernatants from *L. casei* and *L. casei* Δ*pyrR*. The housekeeping genes were the *16S rRNA* gene for *S. aureus* and the *gyrA* gene for *S. typhimurium*. The control group consisted of *S. aureus* and *S. typhimurium* supplemented with LB broth. The results showed that both supernatants could significantly downregulate the expression of biofilm-forming genes, such as *cidA*, *icaA*, *dltB*, *agrA*, *srtA* and *sarA* genes in *S. aureus* (Figure 3A). The biofilm formation-related genes analyzed in *S. typhimurium* included *glgC*, *fimF*, *fliF* and *lpfA* (Figure 3B). A heatmap summarizing fold changes across all genes is shown in Figure 3C. The degree of down-regulation after treatment with the supernatant of *L. casei* Δ*pyrR* was reduced compared with that after treatment with the supernatant of wild-type *L. casei*. There was no significant change in the *srtA* transcript level in *S. aureus* under treatment with both supernatants. Melt curve analysis confirmed a single specific amplification product for each primer pair, with no non-specific amplicons or primer dimers detected across all samples, ensuring the reliability and accuracy of the relative gene expression results.

### 3.4. Pyrimidine Metabolic Dysregulation in Lacticaseibacillus casei Attenuates the Anti-Inflammatory Effect in S. typhimurium-Challenged IPEC-J2 Cells

**Figure 4:**
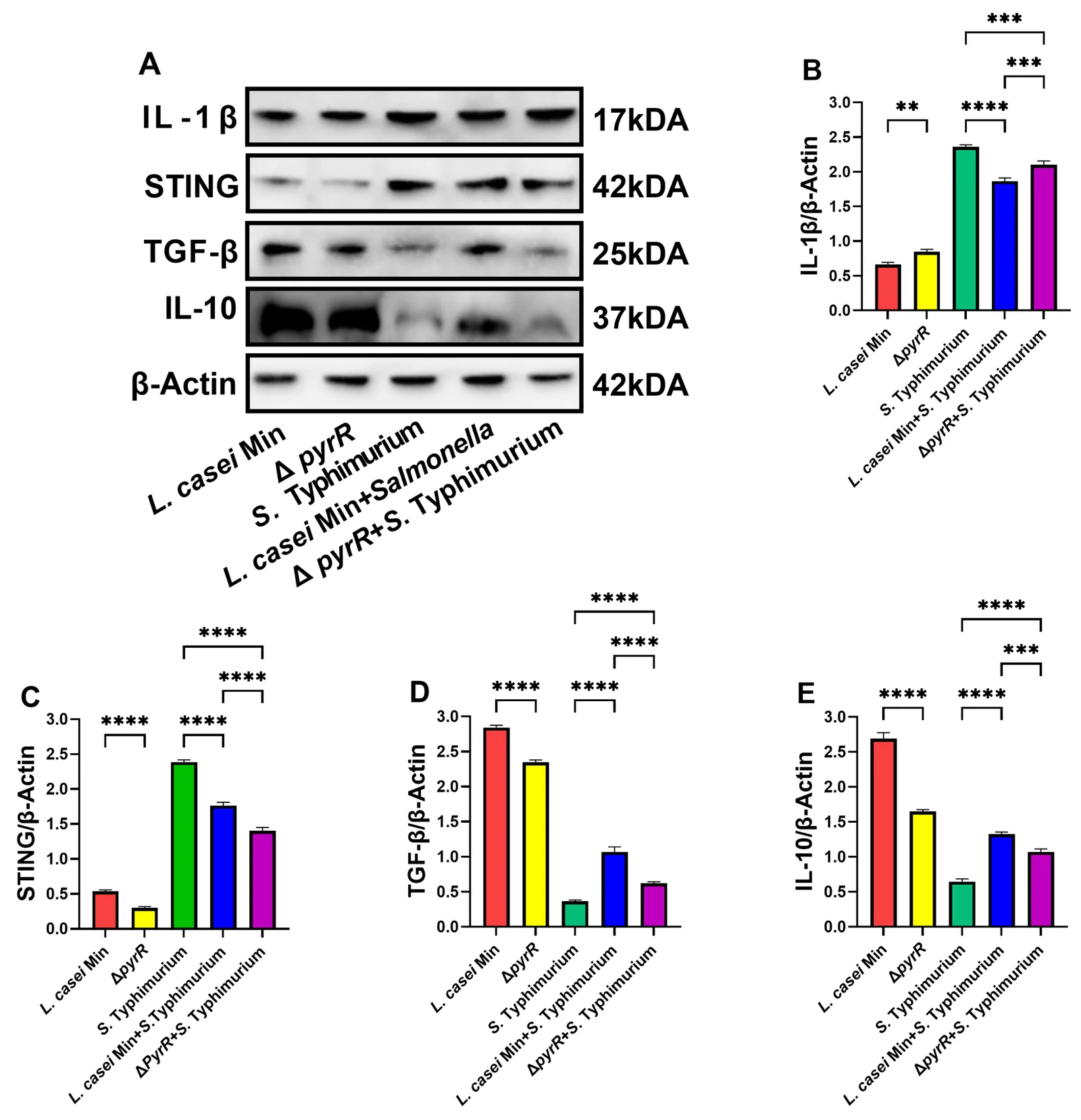
Representative Western blot bands (**A**); quantitative gray-value analysis of relative protein expression levels: mature IL-1β ((**B**), ~17 kDa), STING ((**C**), ~42 kDa), mature TGF-β ((**D**), ~25 kDa), and mature IL-10 ((**E**), ~37 kDa). β-Actin (~42 kDa) was used as the internal reference for normalization. All data are presented as mean ± SD from three independent biological replicates (n = 3). Statistical significance was determined by one-way ANOVA followed by Tukey’s multiple comparison test. **, *p* < 0.01; ***, *p* < 0.001; ****, *p* < 0.0001.

To verify the effect of pyrimidine metabolic dysregulation on the anti-inflammatory capacity, *S. typhimurium* and *L. casei* were used to study the anti-infection effect by examining the expression of related proteins in IPEC-J2 cells (Figure 4A). *Salmonella typhimurium* upregulated IL-1β expression in IPEC-J2 cells, while supernatants of both *L. casei* and *L. casei* Δ*pyrR* downregulated IL-1β expression, even in the presence of *S. typhimurium* (Figure 4B). However, *L. casei* had a significantly greater effect in decreasing the IL-1β expression than *L. casei* Δ*pyrR* (Figure 4B). *L. casei* and *L. casei* Δ*pyrR* decreased the expression level of the stimulator of interferon genes protein (STING) in IPEC-J2 cells. *Salmonella typhimurium* increased the STING expression, and both supernatants from *L. casei* and *L. casei* Δ*pyrR* decreased the STING expression in IPEC-J2 cells co-cultured with *S. typhimurium*. However, STING expression in IPEC-J2 cells treated with the *L. casei* Δ*pyrR* supernatant was significantly lower than that in cells treated with the *L. casei* supernatant (Figure 4C). The existing data only demonstrate an association between *pyrR* status and STING protein abundance, while the underlying regulatory mechanisms and functional significance remain unclear. The supernatants of *L. casei* and *L. casei* Δ*pyrR* could increase the expression level of TGF-β in IPEC-J2. The pathogenic microorganism *S. typhimurium* could reduce the expression of TGF-β. When the supernatants of *L. casei* and *L. casei* Δ*pyrR* were co-cultured with *S. typhimurium*, both of these supernatants increased TGF-β expression in IPEC-J2 cells, inhibiting the inflammatory response. Moreover, the effect of *L. casei* on increasing the expression of TGF-β was significantly better than that of *L. casei* Δ*pyrR*, which indicated that *pyrR*-mediated pyrimidine metabolism contributed to stimulating the expression of TGF-β (Figure 4D). The expression of IL-10 (Figure 4E) in IPEC-J2 cells showed the same trend as TGF-β; both cytokines function to inhibit the inflammatory response.

### 3.5. Pyrimidine Metabolic Dysregulation Attenuates the Capacity of Lacticaseibacillus casei to Inhibit Reactive Oxygen Species Production

**Figure 5:**
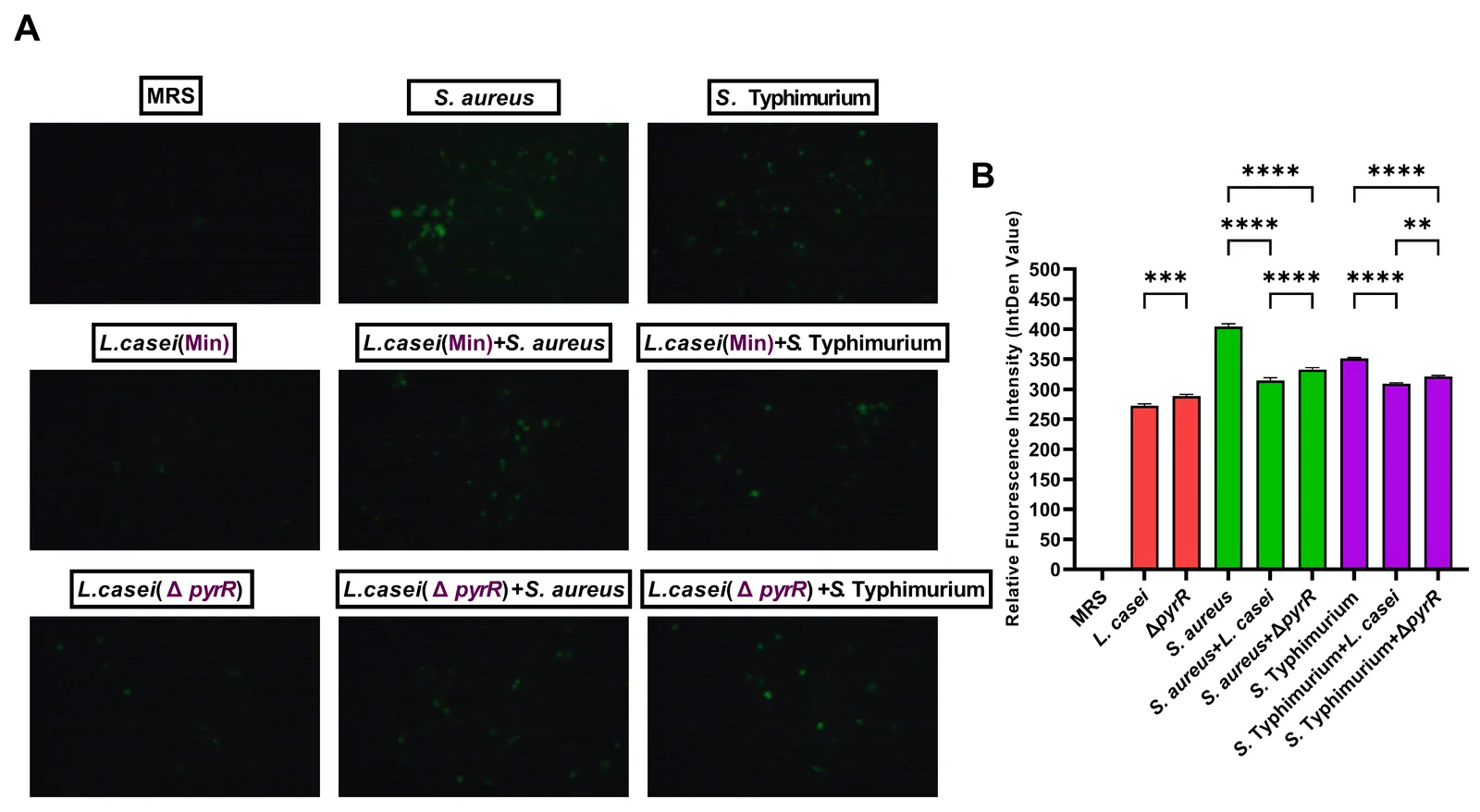
Panel (**A**) shows the fluorescence imaging of ROS under a fluorescence microscope for different groups, and Panel (**B**) presents the quantitative analysis of the relative fluorescence intensity. Green fluorescence corresponds to DCF, the fluorescent oxidation product of DCFH-DA, and indicates the level of intracellular ROS; a brighter green signal indicates higher ROS levels. Experimental groups include *L. casei* (Min) alone, *L. casei* Δ*pyrR* alone, *S. typhimurium* alone, *S. aureus* alone, and co-treatment groups of each *Lactobacillus* supernatant with the two pathogens. All data are presented as mean ± SD from three independent biological replicates (n = 3). Statistical significance was determined by one-way ANOVA followed by Tukey’s multiple comparison test. **, *p* < 0.01; ***, *p* < 0.001; ****, *p* < 0.0001.

Both pathogenic microorganisms, *S. aureus* and *S. typhimurium*, could induce ROS production in IPEC-J2 cells (Figure 5A). The control group was MRS broth. Both *L. casei* and *L. casei* Δ*pyrR* induced a reduction in ROS levels in IPEC-J2 cells (Figure 5B), and *L. casei* exhibited a better inhibitory effect. When co-cultured with pathogenic bacteria, both *L. casei* and *L. casei* Δ*pyrR* significantly suppressed pathogen-induced ROS production in IPEC-J2 cells, and *L. casei* was significantly better than *L. casei* Δ*pyrR* at suppressing ROS production.

## 4. Discussion

LAB can produce antimicrobial substances, such as organic acids and bacteriocins, to inhibit pathogenic microorganisms [32]. Pyrimidines are essential components of organisms with diverse biological functions, and pyrimidine compounds exhibit a broad spectrum of biological activities, including antibacterial, antifungal, antiviral, antimalarial, and antileishmanial properties [33]. Building on our previous finding that *pyrR* knockout impairs the direct bacteriostatic activity of *L. casei* [12], the present study extends this observation by demonstrating that *pyrR*-mediated pyrimidine metabolism also modulates three probiotic functions of the strain: anti-biofilm activity against pathogens, anti-inflammatory activity in host intestinal epithelial cells, and antioxidant capacity against pathogen-induced oxidative stress. LC-MS metabolomics of *L. casei* Δ*pyrR* supernatants confirmed significant reductions in uracil, deoxyadenosine, and pyrimidin-4-amine, alongside decreased secondary antimicrobial metabolites including virginiamycin and L-(+)-lactic acid [12]. These metabolite alterations are functionally relevant, as pyrimidine metabolites are known to modulate biofilm-associated gene networks and the integrity of bacterial membranes in both Gram-positive and Gram-negative pathogens [13,34]. Collectively, our research supports and extends the contribution of pyrimidine metabolism to the probiotic functions of *L. casei*.

Consistent with our previous finding that *pyrR* disruption impairs the antibacterial activity of *L. casei* [12], this study further demonstrates that pyrimidine metabolism also mediates the anti-biofilm, anti-inflammatory and antioxidant functions of this probiotic strain. Inactivation of the *pyrD* gene leads to perturbation of intracellular pyrimidine nucleotide pools in the *Escherichia coli* LF82 strain and strongly affects its biofilm formation ability [35]. *Lactobacillus* strains have also been found to inhibit the biofilms of *S. aureus* and *Salmonella* [36,37]. Our results confirm that disruption of the *pyrR* gene, leading to disturbances in pyrimidine metabolism, impairs the ability of *L. casei* to disrupt pre-formed biofilms of pathogenic microorganisms. Exogenous uracil supplementation can restore the anti-biofilm activity of the supernatant from *L. casei* Δ*pyrR*, but this rescue only partially restores function. The incomplete rescue is not merely a dose effect, but reflects the different levels at which uracil and PyrR act. Exogenous uracil replenishes a single node of the pyrimidine pool through the salvage pathway, whereas PyrR acts upstream as the sensor and transcriptional switch of the entire *pyr* operon. In the Δ*pyrR* strain, loss of PyrR-mediated attenuation uncouples de novo pyrimidine biosynthesis from intracellular pyrimidine availability, so that the operon can no longer be shut off even when pyrimidines are abundant; supplying uracil therefore cannot re-establish this feedback loop and may even coexist with unrestrained de novo synthesis. Furthermore, uracil cannot rebuild the downstream branches derived from the pyrimidine pool, including the secondary antimicrobial metabolites (e.g., virginiamycin and lactic acid) that are depleted in the Δ*pyrR* supernatant. Consequently, the complemented strain remains functionally distinct from the wild-type *L. casei*. During the early stage of biofilm formation in *Salmonella* Enteritidis, the synthesis of extracellular DNA (eDNA) and the regulation of adhesive structures have a high demand for the pyrimidine metabolites uridine, uracil, and thymidine [38]. Pyrimidine metabolites play a crucial role in the production of curli fibers and cellulose, the major components of *Escherichia coli* biofilms, and curli fibers are the primary adhesion factor [39]. Fluoropyrimidines possess antivirulence and anti-biofilm activity against Gram-negative bacteria, inhibiting biofilm formation in *Escherichia coli* and downregulating the transcription of the curli-encoding operon, blocking the production of curli fibers [40]. Our findings indicate that the loss of the *pyrR* gene impairs the anti-biofilm activity of *L. casei* through pyrimidine metabolic dysregulation, which is reflected in weakened morphological damage to pathogens and attenuated modulation of biofilm-associated gene expression.

It should be noted that the cell-free supernatant of *L. casei* is a complex mixture containing multiple antibacterial components, and the attenuated bioactivity of the Δ*pyrR* supernatant results from global metabolic alterations rather than a single metabolite. Among these changes, pyrimidine metabolic dysregulation is the primary effect of *pyrR* gene knockout, as indicated by non-targeted metabolomics experiments from our previous study [12]. The reduction in organic acids such as lactic acid and the accompanying pH change are secondary consequences of impaired pyrimidine metabolism, which further contribute to the overall reduction in antibacterial and anti-biofilm efficacy.

Although treatment with the supernatants from both *L. casei* and *L. casei* Δ*pyrR* resulted in significant downregulation of the expression of biofilm-associated genes *icaA*, *dltB*, *sarA* and *cidA* in *S. aureus*, as well as *glgC*, *fliF*, *lpfA* and *fimF* in *S. typhimurium*, the transcript levels of these genes in the *L. casei* Δ*pyrR* group were significantly higher than those in the *L. casei* group. These selected genes represent the major regulatory pathways affected by *L. casei* pyrimidine metabolites, covering the entire process of biofilm development from initial adhesion to matrix accumulation and maturation. For *S. aureus*, *icaA* mediates the synthesis of polysaccharide intercellular adhesin (PIA), the core polysaccharide component of the biofilm matrix; *agrA* and *sarA* are central quorum-sensing regulators that globally govern biofilm formation; *srtA* anchors surface adhesion proteins to the cell wall; *cidA* controls eDNA release by regulating bacterial cell death; and *dltB* modulates cell surface charge to affect biofilm adhesion. For *S. typhimurium*, *glgC* participates in exopolysaccharide biosynthesis, while *fimF*, *lpfA* and *fliF* are responsible for fimbrial and flagellar assembly, which are critical for early adhesion and three-dimensional biofilm structure formation. The polyhalogenated pyrimidine compound 2,4-dichloro-5-fluoropyrimidine can significantly downregulate the gene expression of *agrA* (quorum sensing regulator), *hla* (α-hemolysin), *nuc1* (nuclease 1), *RNAIII* (quorum sensing regulator), and *saeR* (virulence regulator) in *S. aureus* [41]. Pyrimidinedione can inhibit the activity of DNA adenine methyltransferase (Dam) in *Streptococcus pneumoniae*, down-regulate the expression of biofilm-related genes in *Streptococcus pneumoniae*, and disrupt the structure and biofilm-forming ability of *Streptococcus pneumoniae* [42].

We are aware that the crystal violet biofilm assay measures total adherent biomass and does not separate biofilm-specific disruption from general antibacterial activity by itself, particularly because the wild-type *L. casei* supernatant also possesses stronger antibacterial activity [12]. Nevertheless, two observations in the present study support an anti-biofilm-specific component beyond mere growth inhibition. The RT-qPCR results showed that the *L. casei* supernatant selectively downregulated biofilm- and flagella-associated genes governing matrix synthesis (*icaA*, *glgC*), quorum sensing (*agrA*, *sarA*), and adhesion and flagellar assembly (*fimF*, *fliF*, *lpfA*), and these pathway-specific transcriptional modulations were attenuated by the *L. casei* Δ*pyrR* supernatant; such transcriptional changes are not expected from growth inhibition alone. Exogenous uracil supplementation significantly restored the anti-biofilm activity of the *L. casei* Δ*pyrR* supernatant (Figure 1), providing functional evidence that pyrimidine-related metabolites contribute to the anti-biofilm activity; the incomplete rescue further implies additional contributions from other supernatant components.

Our results indicate that *pyrR*-mediated pyrimidine metabolism supports the probiotic functions of *L. casei* by influencing the inflammatory response and oxidative stress balance in host cells, while pyrimidine metabolic dysregulation induced by *pyrR* deletion attenuates these beneficial effects. The *S. typhimurium*-challenged IPEC-J2 cells stimulated the inflammatory response, and the wild-type *L. casei* supernatant treatment had a much better effect than the *L. casei* Δ*pyrR* supernatant treatment in inhibiting inflammatory factor expression. Importantly, pyrimidine-related metabolites from the probiotic strain increased the expression of TGF-β and IL-10. TGF-β is a key anti-inflammatory cytokine, and its reduced expression attenuates the inhibition of pro-inflammatory factors, further amplifying the inflammatory response. Pyrimidinedione derivatives (biemamides A–E) act as TGF-β pathway inhibitors, exerting their effects by blocking TGF-β-induced epithelial-to-mesenchymal transition (EMT) [43]. It should be clarified that the STING-mediated innate immune pathway exerts bidirectional regulatory effects: excessive STING activation aggravates inflammatory injury, while basal STING signaling is essential for host antibacterial immunity [44,45]. The significant downregulation of STING protein in the *L. casei* Δ*pyrR* group, together with the overall attenuated anti-biofilm, anti-inflammatory and antioxidant phenotypes of the mutant strain, suggests that this STING reduction reflects impaired host innate immune defense rather than an enhanced anti-inflammatory effect. This study detected only the total protein level of STING without examining its downstream activation markers (phospho-IRF3, IFN-β, or cGAS activity). The current data support an association between *pyrR* status and STING abundance, but they do not establish whether this alteration is beneficial, detrimental, or mechanistically responsible for the anti-inflammatory phenotype.

In addition, pyrimidine metabolic dysregulation in *L. casei* Δ*pyrR* attenuated the ability of its supernatant to inhibit ROS generation in IPEC-J2 cells. Pathogenic microorganisms such as *S. typhimurium* and *S. aureus* can induce IPEC-J2 cells to produce ROS. Excessive accumulation of ROS not only damages cells but also disrupts cellular DNA and activates pro-inflammatory signaling pathways, intensifying the inflammatory response and reducing the cells’ ability to resist pathogenic microorganisms [46]. Notably, pyrimidine metabolites regulate mitochondrial pyruvate oxidation and the tricarboxylic acid (TCA) cycle by modulating pyruvate dehydrogenase (PDH) activity [47]. Pyrimidine deficiency compromises mitochondrial pyruvate oxidation efficiency, disrupting mitochondrial energy metabolism accordingly [47]. The depletion of pyrimidine metabolites in the *L. casei* Δ*pyrR* supernatant may impair mitochondrial function, which could be a potential factor contributing to its reduced ability to inhibit pathogenic microorganisms, although this hypothesis requires further experimental validation. We acknowledge that the DCFH-DA fluorescence microscopy method has limited specificity and may be influenced by cell density and cell viability; additional validation using flow cytometry, plate-based fluorescence measurement, or oxidative stress biomarkers is planned for our future studies.

Our study validated the regulatory role of *pyrR* gene-mediated pyrimidine metabolism in the probiotic functions of *L. casei* through genome editing. In terms of morphological analysis, although transmission electron microscopy can directly visualize ultrastructural damage in pathogenic bacteria, complementary quantitative assessments of membrane integrity—such as extracellular ATP detection, propidium iodide uptake analysis, or total protein leakage measurement—can provide objective, quantifiable evidence to support the membrane disruption phenotypes observed under transmission electron microscopy. In addition, the biofilm assay did not include viable cell enumeration or normalization of biofilm biomass to bacterial growth; although the biofilm-gene expression data support an anti-biofilm-specific component, future assays performed under sub-MIC conditions with growth normalization are warranted to directly discriminate biofilm-specific inhibition from general antibacterial effects. To exclude secondary effects arising from CRISPR-mediated genome editing, genetic complementation of the *pyrR* gene (Δ*pyrR*^+^ revertant strain), the definitive test of causality, will be the focus of our subsequent work. A limitation of this study is the absence of in vivo validation. The IPEC-J2 cell line used in this work is a classic and appropriate in vitro model for assessing the response of porcine intestinal epithelial cells to probiotics and pathogens. However, as a two-dimensional monolayer culture system, IPEC-J2 lacks key structural and cellular features of native intestinal tissue, including three-dimensional mucosal architecture, a continuous protective mucus layer, and tissue-resident immune cell populations. The in vivo intestinal environment involves more complex regulatory systems, including the complete gut microbiota, mucosal immune network, intestinal barrier structure, and systemic immune circulation, all of which cannot be fully recapitulated in a monolayer cell culture system. Although the low treatment concentration (10%, *v*/*v*, of pH-neutralized supernatant) and the stimulus-specific protein and ROS responses argue against major treatment-related cytotoxicity, direct cell viability assessment was not performed and will be included in our follow-up studies. These factors may lead to differences between in vitro cellular phenotypes and actual in vivo functional effects. Therefore, the regulatory role of *pyrR*-mediated pyrimidine metabolism in the anti-biofilm, anti-inflammatory and antioxidant functions of *L. casei* remains to be further confirmed in suitable animal models (such as murine or piglet models) in subsequent studies.

## 5. Conclusions

In this study, we investigated whether *pyrR*-mediated pyrimidine metabolism is associated with the probiotic functions of *Lacticaseibacillus casei*. Compared with the wild-type *L. casei* strain, the Δ*pyrR* mutant showed consistently attenuated anti-biofilm and antibacterial activities against *S. aureus* and *S. typhimurium*, together with impaired anti-inflammatory and antioxidant effects in *S. typhimurium*-challenged IPEC-J2 cells. The partial restoration of anti-biofilm activity by exogenous uracil supplementation further linked these functional defects to pyrimidine metabolic dysregulation. Taken together, the current data support an association between *pyrR* deletion-related pyrimidine metabolic dysregulation and impaired probiotic functions of *L. casei*; however, a causal relationship cannot be established from the present data. Genetic complementation and in vivo studies remain necessary to definitively establish causality.

## Figures and Tables

**Table 1:** Primers for *S. aureus* and *S. typhimurium* used in this study.

Gene	Primer	Primer Sequence (5′–3′)
*cidA*	cidA-F	AGCGTAATTTCGGAAGCAACATCCA
	cidA-R	CCCTTAGCCGGCAGTATTGTTGGTC
*dltB*	dltB-F	GTGGACATCAGATTCACTTCC
	dltB-R	ATAGAACCATCACGAATTTCC
*icaA*	icaA-F	GGCTGCGGTAACTGGCAATCC
	icaA-R	CTTGCCAGTTAAAGATTGGGC
*sarA*	sarA-F	TTTCTCTTTGTTTTCGCTGATG
	sarA-R	TTGCTTTGAGTTGTTATCAATGG
*agrA*	agrA-F	CCTCGCAACTGATAATCCTTATG
	agrA-R	ACGAATTTCACTGCCTAATTTGA
*srtA*	srtA-F	CACAGAAGCGGTGATGAA
	srtA-R	AAATAAGGAAACGCCATC
*16S rRNA gene*	16S rRNA-F	GCTGCCCTTTGTATTGTC
	16S rRNA-R	AGATGTTGGGTTAAGTCCC
*glgC*	glgC-F	TGACCAGAACTGGCCTATCC
	glgC-R	CAGCGAGTTCAGCGTCATAC
*fimF*	fimF-F	CCTAACACGTTACCGCTTCA
	fimF-R	CCGGCGTCATATTTTCTGA
*fliF*	fliF-F	GAAGCCATTCTGTCGCCTAT
	fliF-R	TGTAGTGCTCTTCCGTCTGC
*lpfA*	lpfA-F	GATGCGACACTGGTTTCTGT
	lpfA-R	CGGTGTTCATTTCAACAAGC
*gyrA*	gyrA-F	CCAATACGTTCATGGCGTAAAG
	gyrA-R	GATTATGCGATGTCGGTCATTGT

## Data Availability

The authors confirm that the data supporting the findings of this study are available within the manuscript and tables.

## Supplementary Materials

The following supporting information can be downloaded at: https://www.mdpi.com/article/10.3390/biology15211914/s1, Figure S1: Original transmission electron microscopy images of *S. aureus* and *S. typhimurium* after co-culture with cell-free supernatants of *L. casei* and *L. casei* Δ*pyrR*; Figure S2: Original Western blot images; Table S1: OD_590_ values underlying the biofilm biomass heatmap shown in Figure 1.

## Abbreviations

The following abbreviations are used in this manuscript:

*L. casei*	*Lacticaseibacillus casei*
*S. aureus*	*Staphylococcus aureus*
*S. typhimurium*	*Salmonella typhimurium*
RT-qPCR	reverse transcription quantitative real-time polymerase chain reaction
IPEC-J2	porcine jejunal epithelial cells

## References

[1] Smokvina T., Wels M., Polka J., Chervaux C., Brisse S., Boekhorst J., van Hylckama Vlieg J.E., Siezen R.J. (2013). *Lactobacillus paracasei* comparative genomics: Towards species pan-genome definition and exploitation of diversity. PLoS ONE.

[2] Qin D., Bai Y., Li Y., Huang Y., Li L., Wang G., Qu Y., Wang J., Yu L.Y., Hou X. (2022). Changes in Gut Microbiota by the *Lactobacillus casei* Anchoring the K88 Fimbrial Protein Prevented Newborn Piglets from Clinical Diarrhea. Front. Cell. Infect. Microbiol..

[3] Wang Y., Yan X., Zhang W., Liu Y., Han D., Teng K., Ma Y. (2019). *Lactobacillus casei* Zhang Prevents Jejunal Epithelial Damage to Early-Weaned Piglets Induced by *Escherichia coli* K88 via Regulation of Intestinal Mucosal Integrity, Tight Junction Proteins and Immune Factor Expression. J. Microbiol. Biotechnol..

[4] Heredia-Castro P.Y., Méndez-Romero J.I., Hernández-Mendoza A., Acedo-Félix E., González-Córdova A.F., Vallejo-Cordoba B. (2015). Antimicrobial activity and partial characterization of bacteriocin-like inhibitory substances produced by *Lactobacillus* spp. isolated from artisanal Mexican cheese. J. Dairy Sci..

[5] Peng H., Zhou G., Yang X.M., Chen G.J., Chen H.B., Liao Z.L., Zhong Q.P., Wang L., Fang X., Wang J. (2022). Transcriptomic Analysis Revealed Antimicrobial Mechanisms of *Lactobacillus rhamnosus* SCB0119 against *Escherichia coli* and *Staphylococcus aureus*. Int. J. Mol. Sci..

[6] Snell E.E., Mitchell H.K. (1941). Purine and Pyrimidine as Growth Substances for Lactic Acid Bacteria. Proc. Natl. Acad. Sci. USA.

[7] Wang J., Wu R., Zhang W., Sun Z., Zhao W., Zhang H. (2013). Proteomic comparison of the probiotic bacterium *Lactobacillus casei* Zhang cultivated in milk and soy milk. J. Dairy Sci..

[8] Li L., Zhang H., Meng D., Yin H. (2023). Transcriptomics of *Lactobacillus paracasei*: Metabolism patterns and cellular responses under high-density culture conditions. Front. Bioeng. Biotechnol..

[9] Jørgensen C.M., Fields C.J., Chander P., Watt D., Burgner J.W., Smith J.L., Switzer R.L. (2008). pyr RNA binding to the *Bacillus caldolyticus* PyrR attenuation protein-characterization and regulation by uridine and guanosine nucleotides. FEBS J..

[10] Turnbough C.L., Switzer R.L. (2008). Regulation of pyrimidine biosynthetic gene expression in bacteria: Repression without repressors. Microbiol. Mol. Biol. Rev..

[11] Turner R.J., Lu Y., Switzer R.L. (1994). Regulation of the *Bacillus subtilis* pyrimidine biosynthetic (pyr) gene cluster by an autogenous transcriptional attenuation mechanism. J. Bacteriol..

[12] Chen S., He X., Qin Z., Li G., Wang W., Nai Z., Tian Y., Liu D., Jiang X. (2023). Loss in the Antibacterial Ability of a PyrR Gene Regulating Pyrimidine Biosynthesis after Using CRISPR/Cas9-Mediated Knockout for Metabolic Engineering in *Lactobacillus casei*. Microorganisms.

[13] Barkat Ali K., Muhammad Azhar Ud D., Mohammed Talab M., Maimoona B., Habib U. (2025). Antagonism and Antibiofilm Activity of Sterile Microbiota Growth Medium against *Klebsiella pneumoniae* In Vitro. World J. Exp. Biosci..

[14] Chen J., Pang H., Wang L., Ma C., Wu G., Liu Y., Guan Y., Zhang M., Qin G., Tan Z. (2022). Bacteriocin-Producing Lactic Acid Bacteria Strains with Antimicrobial Activity Screened from Bamei Pig Feces. Foods.

[15] Giordani B., Naldi M., Croatti V., Parolin C., Erdoğan Ü., Bartolini M., Vitali B. (2023). Exopolysaccharides from vaginal lactobacilli modulate microbial biofilms. Microb. Cell Factories.

[16] Abruzzo A., Giordani B., Parolin C., De Gregorio P.R., Foschi C., Cerchiara T., Bigucci F., Vitali B., Luppi B. (2021). *Lactobacillus crispatus* BC1 Biosurfactant Delivered by Hyalurosomes: An Advanced Strategy to Counteract Candida Biofilm. Antibiotics.

[17] Wallis J.K., Krömker V., Paduch J.H. (2018). Biofilm formation and adhesion to bovine udder epithelium of potentially probiotic lactic acid bacteria. AIMS Microbiol..

[18] Ball A.L., Augenstein E.D., Wienclaw T.M., Richmond B.C., Freestone C.A., Lewis J.M., Thompson J.S., Pickett B.E., Berges B.K. (2022). Characterization of *Staphylococcus aureus* biofilms via crystal violet binding and biochemical composition assays of isolates from hospitals, raw meat, and biofilm-associated gene mutants. Microb. Pathog..

[19] Saidi N., Saderi H., Owlia P., Soleimani M. (2023). Anti-Biofilm Potential of *Lactobacillus casei* and *Lactobacillus rhamnosus* Cell-Free Supernatant Extracts against *Staphylococcus aureus*. Adv. Biomed. Res..

[20] Naghili H., Tajik H., Raeisi M., Ghasem H., Moradi M., Aminzare M., Mehdizadeh T., Hasanzadazar H., Hariri F. (2018). Inhibitory Potential of Metabolites from *Lactobacillus casei* against Phenotypic Characteristics of *Salmonella typhimurium* LT2. Med. Lab. J..

[21] Yang X., Peng Z., He M., Li Z., Fu G., Li S., Zhang J. (2024). Screening, probiotic properties, and inhibition mechanism of a *Lactobacillus* antagonistic to *Listeria monocytogenes*. Sci. Total Environ..

[22] Jiang X., Yan X., Gu S., Yang Y., Zhao L., He X., Chen H., Ge J., Liu D. (2019). Biosurfactants of *Lactobacillus helveticus* for biodiversity inhibit the biofilm formation of *Staphylococcus aureus* and cell invasion. Future Microbiol..

[23] Campos-Galvão M.E., Ribon A.O., Araújo E.F., Vanetti M.C. (2016). Changes in the *Salmonella enterica* Enteritidis phenotypes in presence of acyl homoserine lactone quorum sensing signals. J. Basic Microbiol..

[24] Yan X., Gu S., Cui X., Shi Y., Wen S., Chen H., Ge J. (2019). Antimicrobial, anti-adhesive and anti-biofilm potential of biosurfactants isolated from *Pediococcus acidilactici* and *Lactobacillus plantarum* against *Staphylococcus aureus* CMCC26003. Microb. Pathog..

[25] Arocho A., Chen B., Ladanyi M., Pan Q. (2006). Validation of the 2-DeltaDeltaCt calculation as an alternate method of data analysis for quantitative PCR of BCR-ABL P210 transcripts. Diagn. Mol. Pathol. Am. J. Surg. Pathol. Part B.

[26] Brosnahan A.J., Brown D.R. (2012). Porcine IPEC-J2 intestinal epithelial cells in microbiological investigations. Vet. Microbiol..

[27] Palkovicsné Pézsa N., Kovács D., Somogyi F., Karancsi Z., Móritz A.V., Jerzsele Á., Rácz B., Farkas O. (2023). Effects of *Lactobacillus rhamnosus* DSM7133 on Intestinal Porcine Epithelial Cells. Animals.

[28] Schierack P., Kleta S., Tedin K., Babila J.T., Oswald S., Oelschlaeger T.A., Hiemann R., Paetzold S., Wieler L.H. (2011). *E. coli* Nissle 1917 Affects *Salmonella* adhesion to porcine intestinal epithelial cells. PLoS ONE.

[29] Wang W., Han F., He X., Zhao S., Zou Z., Tian M., Chen H., Wu S., Sun Y., Jiang Y. (2025). Breed Varieties of Pigs for Disease Resistance and Susceptibility to Seneca Valley Virus Infection. Int. J. Mol. Sci..

[30] Eruslanov E., Kusmartsev S. (2010). Identification of ROS using oxidized DCFDA and flow-cytometry. Methods Mol. Biol..

[31] Kim T.K. (2017). Understanding one-way ANOVA using conceptual figures. Korean J. Anesthesiol..

[32] Banicod R.J.S., Tabassum N., Javaid A., Kim Y.M., Khan F. (2026). Lactic Acid Bacteria-Derived Secondary Metabolites: Emerging Natural Alternatives for Food Preservation. Probiotics Antimicrob. Proteins.

[33] Sharma V., Chitranshi N., Agarwal A.K. (2014). Significance and biological importance of pyrimidine in the microbial world. Int. J. Med. Chem..

[34] Provenzani R., San-Martin-Galindo P., Hassan G., Legehar A., Kallio A., Xhaard H., Fallarero A., Yli-Kauhaluoma J. (2021). Multisubstituted pyrimidines effectively inhibit bacterial growth and biofilm formation of *Staphylococcus aureus*. Sci. Rep..

[35] Rossi E., Leccese G., Baldelli V., Bibi A., Scalone E., Camilloni C., Paroni M., Landini P. (2022). Inactivation of the Pyrimidine Biosynthesis pyrD Gene Negatively Affects Biofilm Formation and Virulence Determinants in the Crohn’s Disease-Associated Adherent Invasive *Escherichia coli* LF82 Strain. Microorganisms.

[36] Ahn K.B., Baik J.E., Yun C.H., Han S.H. (2018). Lipoteichoic Acid Inhibits *Staphylococcus aureus* Biofilm Formation. Front. Microbiol..

[37] Merino L., Trejo F.M., De Antoni G., Golowczyc M.A. (2019). *Lactobacillus* strains inhibit biofilm formation of *Salmonella* sp. isolates from poultry. Food Res. Int..

[38] Guillín Y., Ortiz C., Hidalgo W. (2025). Comparative metabolic study of planktonic and sessile cells in *Salmonella* Enteritidis ATCC 13076: Elucidating metabolic pathways driving biofilm formation. PLoS ONE.

[39] Garavaglia M., Rossi E., Landini P. (2012). The pyrimidine nucleotide biosynthetic pathway modulates production of biofilm determinants in *Escherichia coli*. PLoS ONE.

[40] Ravishankar S., Baldelli V., Angeletti C., Raffaelli N., Landini P., Rossi E. (2024). Fluoropyrimidines affect de novo pyrimidine synthesis impairing biofilm formation in *Escherichia coli*. Biofilm.

[41] Sim M., Kim Y.G., Lee J.H., Lee J. (2024). Antibiofilm Activities of Multiple Halogenated Pyrimidines Against *Staphylococcus aureus*. Int. J. Mol. Sci..

[42] Yadav M.K., Go Y.Y., Chae S.W., Song J.J. (2015). The Small Molecule DAM Inhibitor, Pyrimidinedione, Disrupts *Streptococcus pneumoniae* Biofilm Growth In Vitro. PLoS ONE.

[43] Zhang F., Braun D.R., Ananiev G.E., Hoffmann F.M., Tsai I.W., Rajski S.R., Bugni T.S. (2018). Biemamides A-E, Inhibitors of the TGF-β Pathway That Block the Epithelial to Mesenchymal Transition. Org. Lett..

[44] Liu N., Pang X., Zhang H., Ji P. (2021). The cGAS-STING Pathway in Bacterial Infection and Bacterial Immunity. Front. Immunol..

[45] Guimarães E.S., Marinho F.V., de Queiroz N., Antunes M.M., Oliveira S.C. (2021). Impact of STING Inflammatory Signaling during Intracellular Bacterial Infections. Cells.

[46] Chen A., Huang H., Fang S., Hang Q. (2024). ROS: A “booster” for chronic inflammation and tumor metastasis. Biochim. Biophys. Acta Rev. Cancer.

[47] Sahu U., Villa E., Reczek C.R., Zhao Z., O’Hara B.P., Torno M.D., Mishra R., Shannon W.D., Asara J.M., Gao P. (2024). Pyrimidines maintain mitochondrial pyruvate oxidation to support de novo lipogenesis. Science.

