# Supplementary material for: Pyrimidine Metabolic Dysregulation Following pyrR Knockout Attenuates the Anti-Biofilm, Anti-Inflammatory, and Antioxidant Activities of Lacticaseibacillus casei Cell-Free Supernatant
Source: Biology (Basel). 2026 Oct 22;15(21):1914. doi: 10.3390/biology15211914 (PMC13761523; doi:10.3390/biology15211914)

Determination of the Minimum Inhibitory Concentration (MIC) of the *L. casei* Cell-Free Supernatant against *S. aureus* and *S.* Typhimurium

The MIC of the *L. casei* cell-free supernatant against *S. aureus* and *S*. Typhimurium was determined by the standard broth microdilution method. Briefly, the cell-free supernatant of *L. casei*, prepared as described in Section 2.2, was serially two-fold diluted with sterile Mueller-Hinton broth (MHB) to obtain final supernatant dilution ratios ranging from 1:1 (undiluted) to 1:256 (v/v). A 100-μL aliquot of each dilution was transferred into the wells of a sterile 96-well microplate. Overnight cultures of *S. aureus* and *S*. Typhimurium were adjusted to a 0.5 McFarland standard (1.5×10^8 CFU/mL) and further diluted 1000-fold in MHB to a final density of approximately 1.5×10^5 CFU/mL. A 100-μL aliquot of each bacterial suspension was then added to the corresponding wells, yielding a final volume of 200 μL per well and a final bacterial concentration of approximately 7.5×10^4 CFU/mL. Wells containing MHB and the bacterial inoculum without supernatant served as the growth control, whereas wells containing MHB without bacterial inoculation served as the sterility control. The microplates were incubated aerobically at 37 °C for 24 h. The MIC was defined as the highest dilution ratio of the supernatant that completely inhibited visible bacterial growth after 24 h of incubation, as determined by the absence of visible turbidity. To determine the minimum bactericidal concentration (MBC), 10-μL aliquots from wells showing no visible growth were subcultured onto MHB agar plates and incubated at 37 °C for 24 h; the MBC was defined as the lowest supernatant concentration that yielded no colony growth (i.e., ≥99.9% killing of the initial inoculum). All experiments were performed in triplicate with three independent biological replicates. The resulting MIC, 1/2 MIC, and 1/4 MIC of the *L. casei* supernatant against each pathogen were subsequently used in the TEM morphological analysis (Section 2.4).

The MICs of the *L. casei* cell-free supernatant against *S. aureus* and *S*. Typhimurium were determined to be 1:2 and 1:4 (v/v), respectively, with corresponding MBCs of 1:1 (undiluted) and 1:2 (v/v). *S*. Typhimurium was more sensitive to the *L. casei* supernatant than *S. aureus*, consistent with previous reports demonstrating that the antibacterial metabolites of *L. casei* are more effective against enteric Gram-negative pathogens. These MIC values were used to establish the treatment concentrations (MIC, 1/2 MIC, and 1/4 MIC) applied in the subsequent TEM morphological analysis.

Original images of western blot:

After confirming the molecular weights of target proteins via preliminary experiments, we trimmed the PVDF membranes before primary antibody incubation to conserve antibody reagents and consumables.

IL-1: From left to right, they are: *L. casei* group, *L. casei* Δ*pyrR* group, *Salmonella* group, *L. casei* co-cultured with *Salmonella* group and *L. casei* Δ*pyrR* co-cultured with *Salmonella* group.

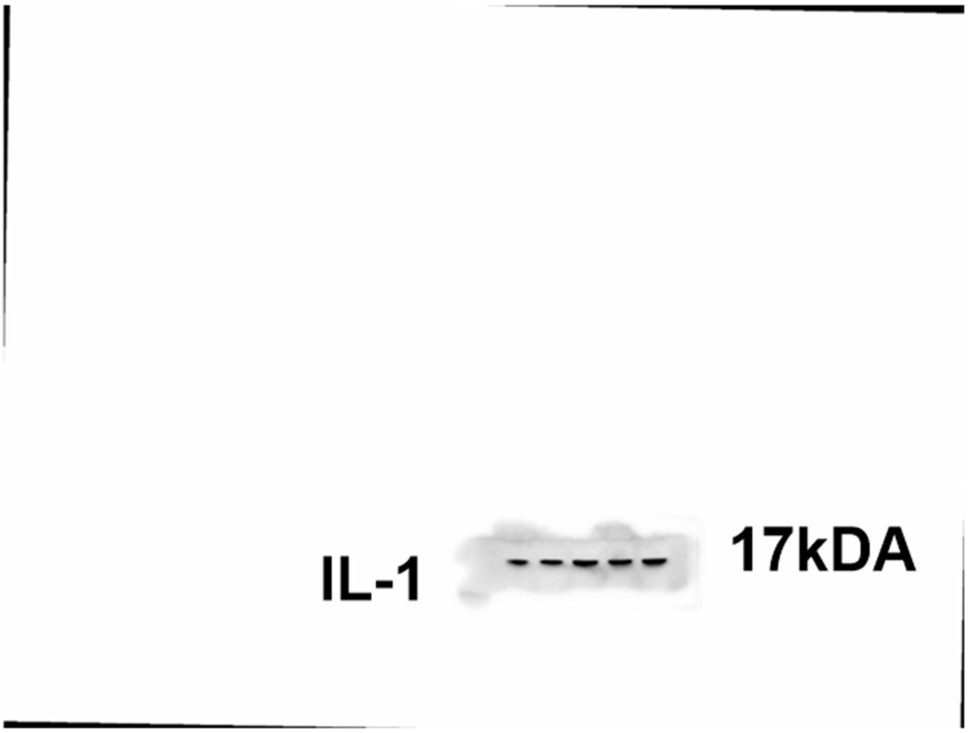
IL-1
17kDA

STING: From left to right, they are: *L. casei* group, *L. casei* Δ*pyrR* group, *Salmonella* group, *L. casei* co-cultured with *Salmonella* group and *L. casei* Δ*pyrR* co-cultured with *Salmonella* group.

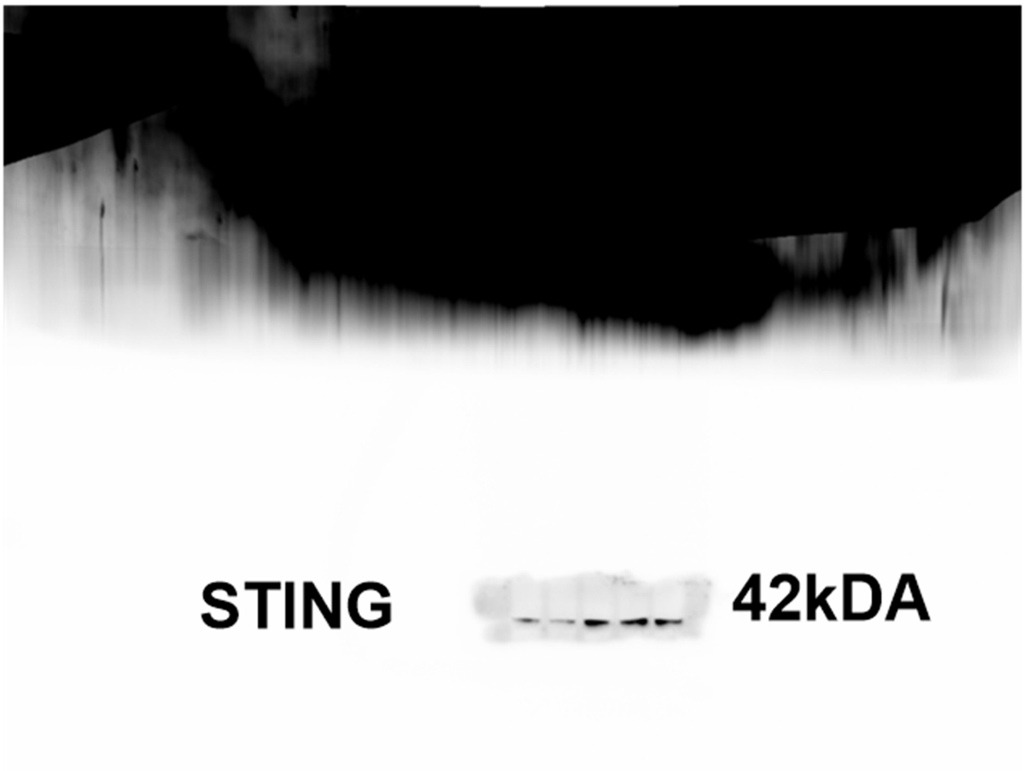

TGF-β: From left to right, they are: *L. casei* group, *L. casei* Δ*pyrR* group, *Salmonella* group, *L. casei* co-cultured with *Salmonella* group and *L. casei* Δ*pyrR* co-cultured with *Salmonella* group.

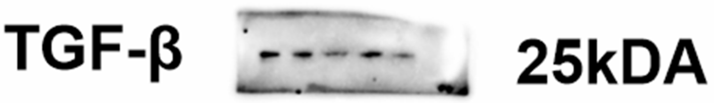

IL-10: From left to right, they are: *L. casei* group, *L. casei* Δ*pyrR* group, *Salmonella* group, *L. casei* co-cultured with *Salmonella* group and *L. casei* Δ*pyrR* co-cultured with *Salmonella* group.

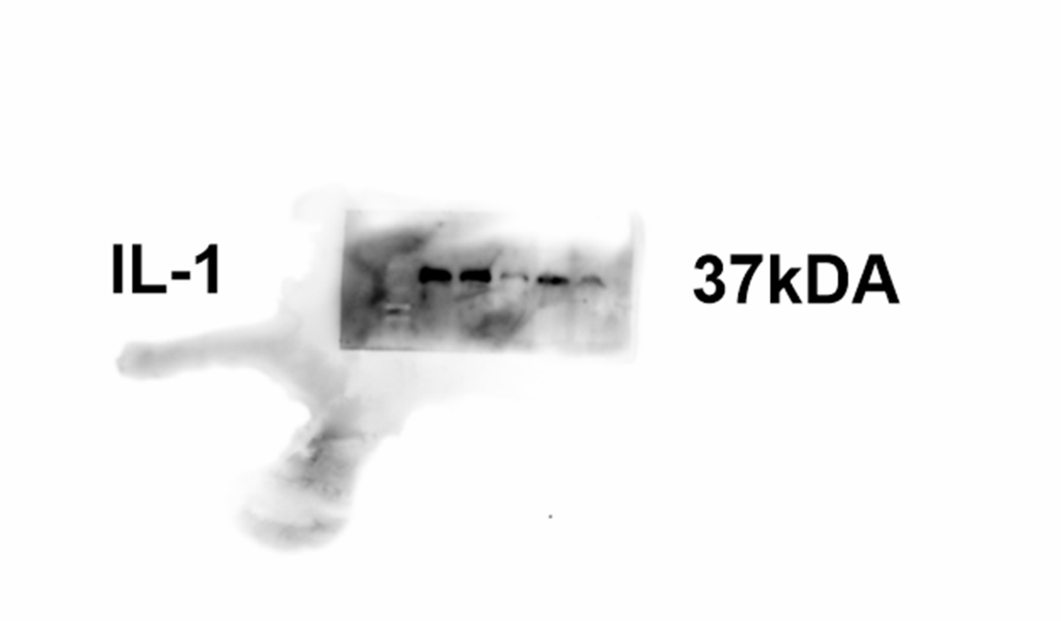

β-Actin: From left to right, they are: *L. casei* group, *L. casei* Δ*pyrR* group, *Salmonella* group, *L. casei* co-cultured with *Salmonella* group and *L. casei* Δ*pyrR* co-cultured with *Salmonella* group.

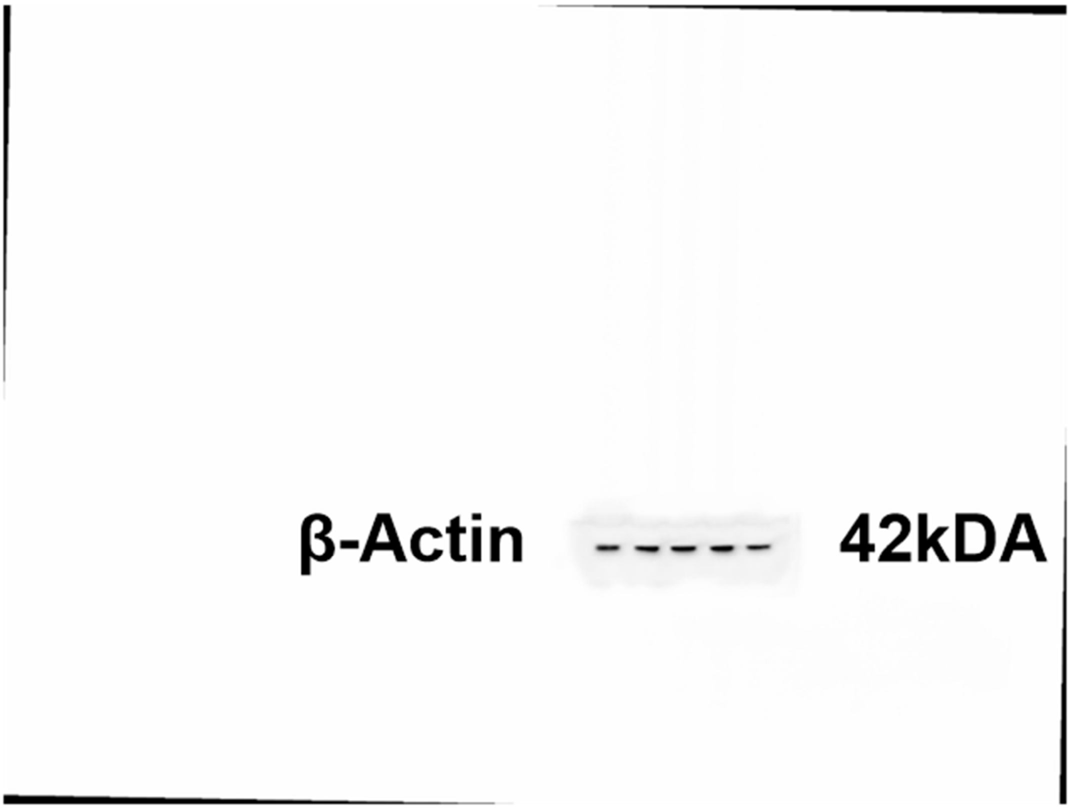

Supplement: Supplementary file 1 [file biology-15-01914-s001.zip › Supplementary content.pdf]
