# Supplementary material for: Pyrimidine Metabolic Dysregulation Following pyrR Knockout Attenuates the Anti-Biofilm, Anti-Inflammatory, and Antioxidant Activities of Lacticaseibacillus casei Cell-Free Supernatant
Source: Biology (Basel). 2026 Oct 22;15(21):1914. doi: 10.3390/biology15211914 (PMC13761523; doi:10.3390/biology15211914)

Original images of western blot:

After confirming the molecular weights of target proteins via preliminary experiments, we trimmed the PVDF membranes before primary antibody incubation to conserve antibody reagents and consumables.

IL-1: From left to right, they are: *L. casei* group, *L. casei* Δ*pyrR* group, *Salmonella* group, *L. casei* co-cultured with *Salmonella* group and *L. casei* Δ*pyrR* co-cultured with *Salmonella* group.

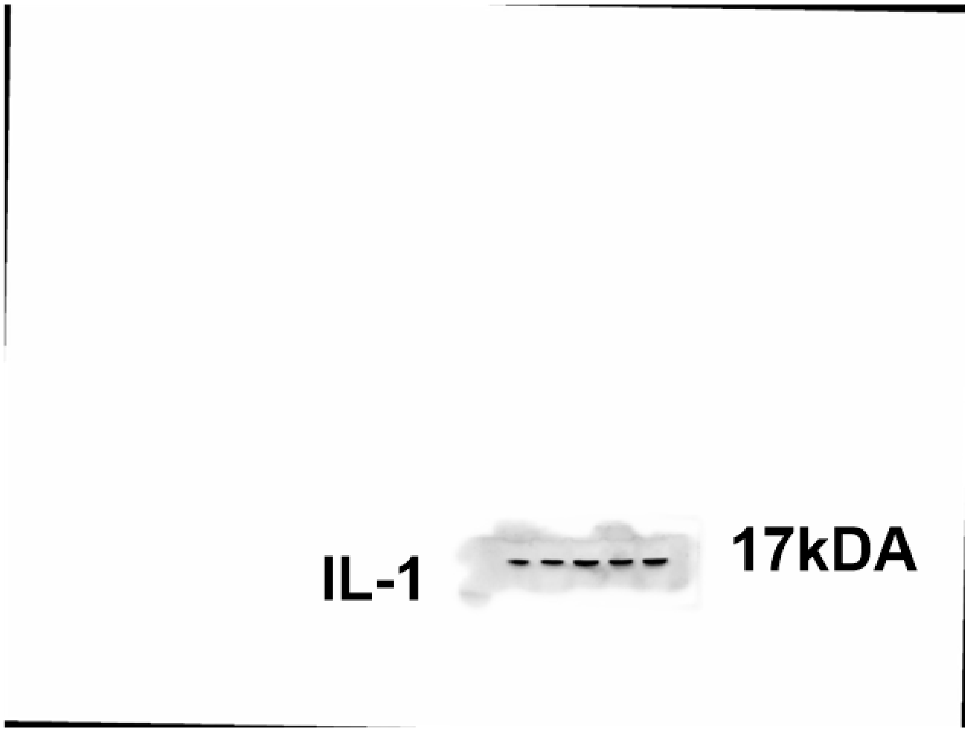

STING: From left to right, they are: *L. casei* group, *L. casei* Δ*pyrR* group, *Salmonella* group, *L. casei* co-cultured with *Salmonella* group and *L. casei* Δ*pyrR* co-cultured with *Salmonella* group.

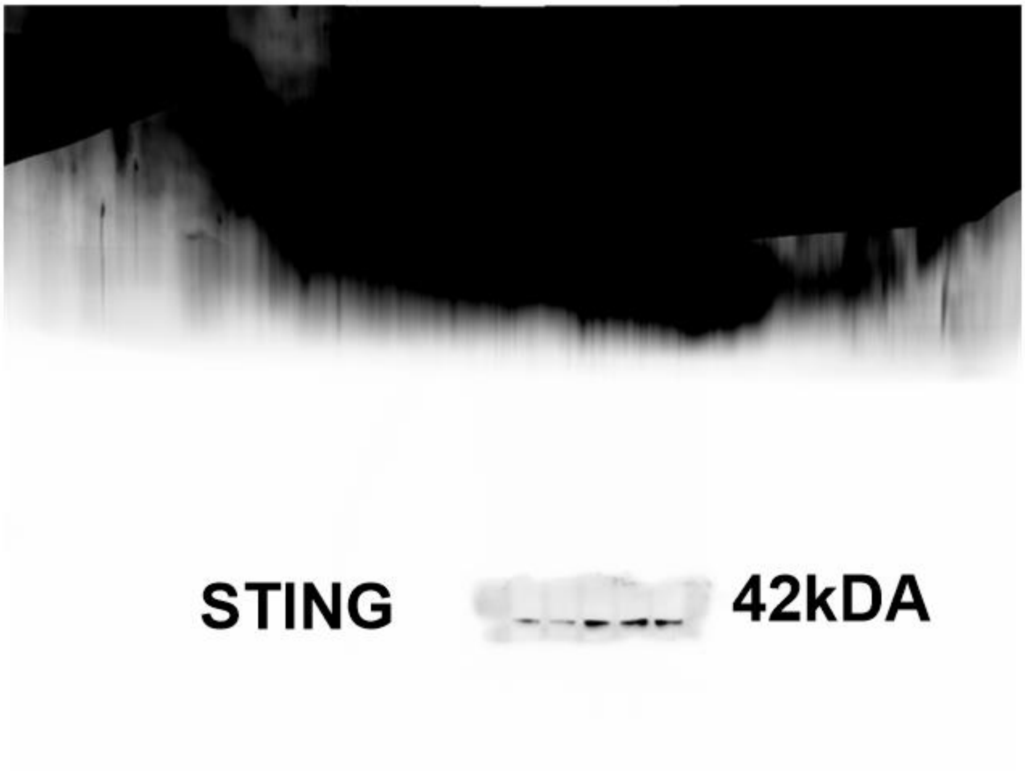

TGF-β: From left to right, they are: *L. casei* group, *L. casei* Δ*pyrR* group, *Salmonella* group, *L. casei* co-cultured with *Salmonella* group and *L. casei* Δ*pyrR* co-cultured with *Salmonella* group.

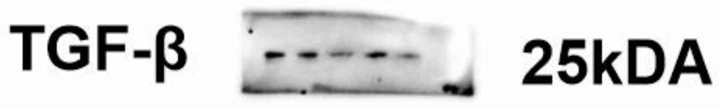

IL-10: From left to right, they are: *L. casei* group, *L. casei* Δ*pyrR* group, *Salmonella* group, *L. casei* co-cultured with *Salmonella* group and *L. casei* Δ*pyrR* co-cultured with *Salmonella* group.

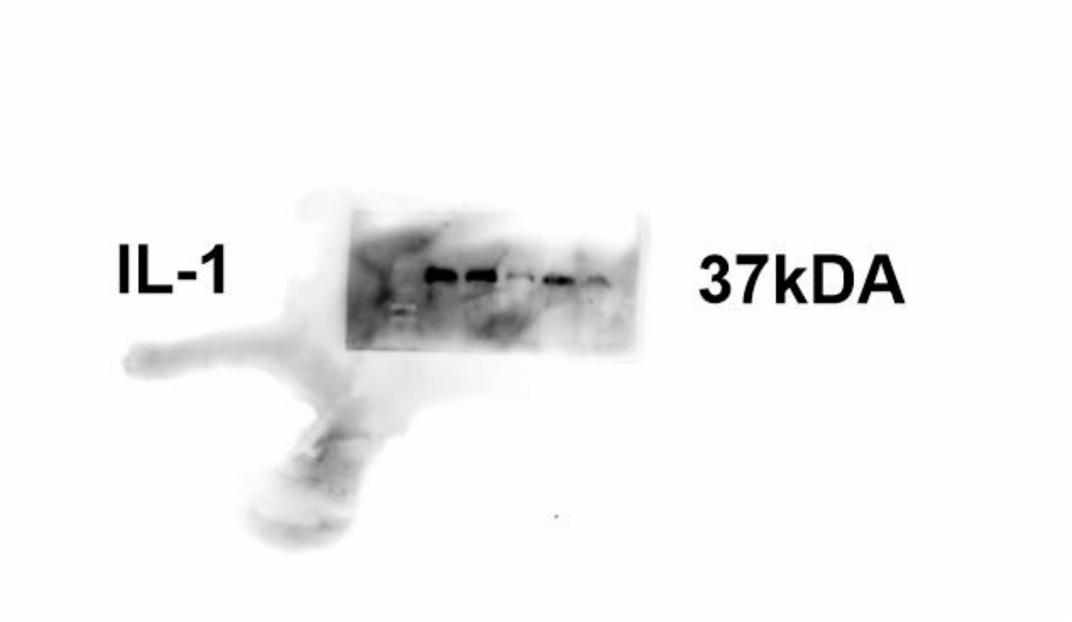

β-Actin: From left to right, they are: *L. casei* group, *L. casei* Δ*pyrR* group, *Salmonella* group, *L. casei* co-cultured with *Salmonella* group and *L. casei* Δ*pyrR* co-cultured with *Salmonella* group.

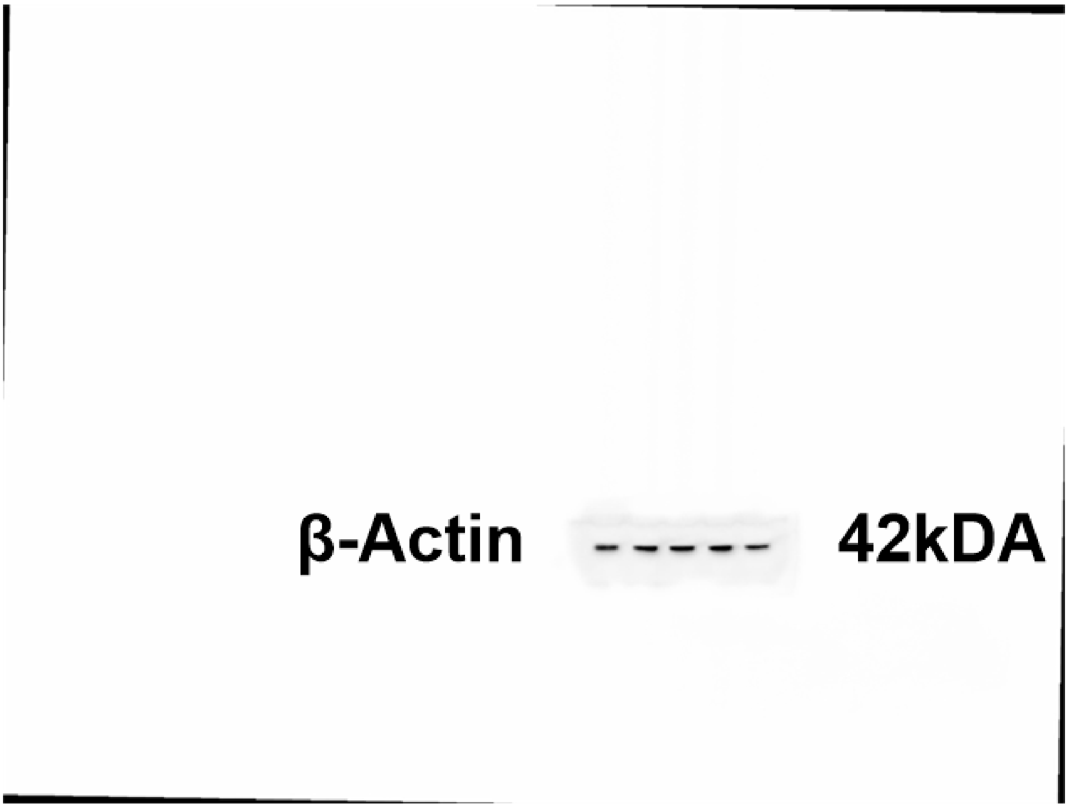

Supplement: Supplementary file 1 [file biology-15-01914-s001.zip › Figure S2 (Western blot)/Original images of western blot (1).pdf]
